# Effects of Concrete Grades and Column Spacings on the Optimal Design of Reinforced Concrete Buildings

**DOI:** 10.3390/ma15124290

**Published:** 2022-06-17

**Authors:** Mohammed Rady, Sameh Youssef Mahfouz

**Affiliations:** Construction and Building Engineering Department, College of Engineering and Technology, Arab Academy for Science, Technology and Maritime Transport (AASTMT), B 2401 Smart Village, Giza 12577, Egypt; symahfouz@aast.edu

**Keywords:** evolutionary, floor systems, slenderness, excel solver, tuning, structural design

## Abstract

This paper investigates the effects of concrete grades and column spacings on the optimal design of reinforced concrete (RC) buildings. To this end, cost design optimization was performed for buildings with three different floor systems: flat plates (FS), flat slabs with drop panels (FSDP), and solid slabs (SS). The evolutionary method provided by the Excel solver was used as the optimization algorithm because it can deal with the complex nature of structural design problems. The objective function was the total construction cost of the building, including the costs of concrete, reinforcement bars, labor, and formwork, while still fulfilling the constraints of the Egyptian code of practice (ECP-18). The applicability of the presented algorithm was investigated in a design example, where the tuning of the evolutionary algorithm control parameters was sought, and the best parameters were investigated. Two case studies were employed to study the impacts of changing the column spacing and concrete grades on the optimal cost for each floor system. The results showed that low concrete grades, (i.e., characteristic strength up to 40 MPa) and column spacings up to 5 m are preferred in terms of direct construction costs for low-rise RC residential buildings.

## 1. Introduction

The construction costs of materials are important issues in the structural design of reinforced concrete (RC) structures [1,2,3]. Concrete and steel reinforcement are the main factors affecting direct construction costs [4]. Therefore, it is favorable to consider lighter RC structural components while still meeting the design provisions imposed by the standards and codes [5,6,7,8,9,10].

Despite the efforts of designers to obtain economic cross-sectional dimensions and steel bars of RC members, the conventional design process usually fails to minimize the materials costs [11,12]. This process is based on the prior designer’s experience in selecting the floor system, concrete class, and preliminary cross-sectional dimensions of RC members [3,13]. Most of the designer’s choices are based on rules of thumb to minimize the calculations effort. However, because the process is strictly based on the trial-and-error approach, it is typically expensive in terms of material consumption, computational time, and human effort. Therefore, researchers have been investigating the optimal design of RC members using optimization techniques rather than experience-based procedures [3,14,15].

Generally, the structural design optimization problems are non-linear and involve a high level of complexity [16]. Furthermore, the design variables are discrete and are much dependent on each other. Many researchers used nature-inspired metaheuristic algorithms to generate random design variables using stochastic methods to deal with such problems. These algorithms include artificial bee colony algorithm [3], genetic algorithms [17,18,19,20], simulated annealing [14,21], ant colony optimization [22,23], harmony search [13,24,25], cuckoo search [26], and firefly algorithm [27]. Because the design office practice is mostly based on simple Microsoft Excel spreadsheets, several researchers tested the applicability of the solver tool provided by Microsoft Excel in the field of structural optimization [12,16,28,29,30,31]. The optimization problems in these studies were limited to minimizing the cost of individual structural components such as slabs, beams, footings, and retaining walls.

Many studies discussed the application of environmental, social, and governance objectives in the building sector to achieve sustainable buildings [32,33,34,35]. The efficient structural design could mitigate the excessive waste and environmental impacts of the construction materials, while still lowering the cost of the structure [36,37]. Ženíšek et al. [38] performed an exhaustive search to obtain the optimal solution for RC load-bearing buildings in terms of construction costs and environmental impacts. They studied the effects of two column spacings (4 m and 8 m) and ten concrete characteristic compressive strengths $f_{cu}$ (25–80 MPa) on the optimal costs. The design variables were the column width and the slab thickness. The environmental impacts of different variants during the production of materials, transportation, and construction were discussed. Robati et al. [39] used CSI software programs and Microsoft Excel spreadsheets to design RC office buildings with constant column spacings (5.27 m) following the Australian standards. The authors considered two concrete types (lightweight concrete and ordinary-weight concrete) and two floor systems (waffle slabs and flat slabs). A comparison was held between each design alternative regarding material consumption, CO_2_ emissions, and energy consumption.

To further investigate the effects of column spacings and concrete grades, Rady et al. [40] employed a comparative study of the optimal results of RC residential buildings with three different floor systems. The design variables included the cross-sectional dimensions and steel bars of floors and columns, column spacings, and concrete grades. The authors found that including the column spacings and concrete grades in the design variables can affect the optimal design of different floor systems.

**Figure 1:**
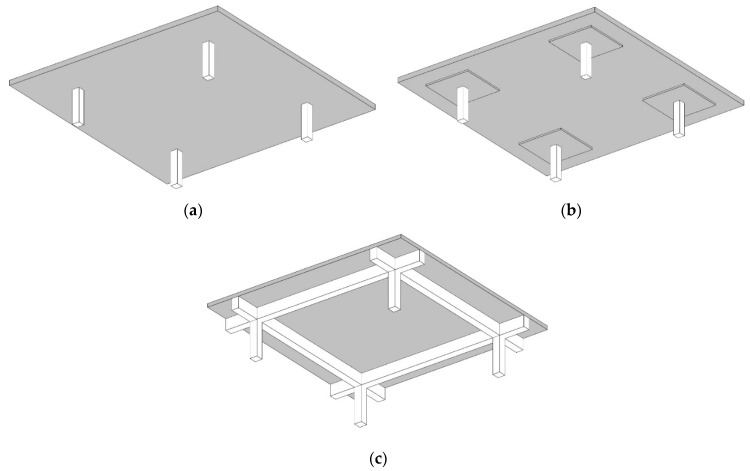
Schemes of buildings with different floor systems: (**a**) flat plates (FS); (**b**) flat slabs with drop panels (FSDP); (**c**) solid slabs (SS).

The current study focuses on minimizing the direct construction costs of low-rise RC residential buildings, taking into account the costs of concrete, steel, labor, and formwork. The main objective of the current study is to investigate the effects of changing the concrete grades and column spacings on the optimal total costs of RC buildings with different floor systems. Thus, three floor systems were optimized: flat plates (FS), flat slabs with drop panels (FSDP), and solid slabs (SS). The building scheme for each system is depicted in Figure 1. The optimization was performed using the Excel solver add-in’s evolutionary algorithm (EA). The design variables, (i.e., the cross-sectional dimensions and the steel bars) were chosen from sets of prescribed discrete values to fulfill the practical requirements. The constraints were the limits regulated by the Egyptian code of practice (ECP-203-18) [41]. Firstly, the authors tuned the EA control parameters to adequately pre-define the best combination of parameters that leads to the best solution in a reasonable time. Secondly, the authors presented two case studies to investigate the effects of the concrete grades and column spacings on the optimal costs of RC buildings with different floor systems. Eight concrete grades and six column spacings were considered in each case study.

The unit prices of materials were obtained from the official periodicals in Egypt, (i.e., the monthly bulletins of building materials average prices provided by the Egyptian ministry of housing). The average labor and formwork unit price was derived from Egypt’s construction sites.

## 2. Design Procedures

In this section, the design procedures of all structural elements, (i.e., slabs, beams, and columns) are presented in accordance with ECP 203-18 [41]. The load patterns, safety factors of dead loads and live loads, and magnitude of live loads were defined by the Egyptian code of loads (ECL) [42].

**Figure 2:**
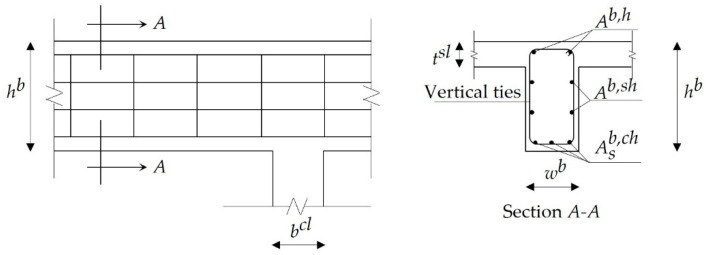
Typical steel bars arrangement for RC beams.

The procedure starts with reading the input data, (i.e., geometry parameters, grades, elastic moduli of materials, etc.). The preliminary cross-sectional dimensions of slabs and beams were determined as per the design code requirements. The design bending moments were calculated at critical cross-sections, the corresponding steel bars were determined, and the maximum deflections were calculated. The punching shear stresses were calculated at slab-column connections and the shear stimulants were evaluated at critical cross-sections of each beam. The ultimate and serviceability limit states were checked to ensure the safety of the slabs and beams. Figure 2 shows the beam cross-sectional dimensions and typical arrangement of longitudinal and transverse steel reinforcement.

**Figure 3:**
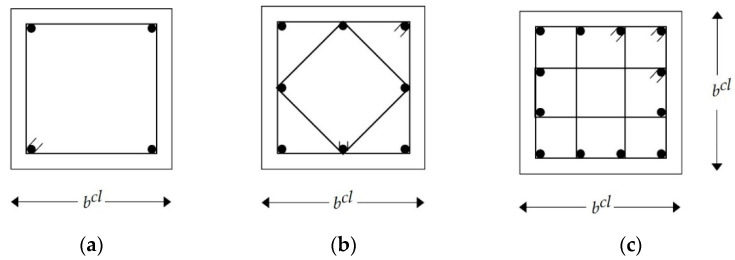
Typical steel bar arrangements for RC columns: (**a**) first arrangement; (**b**) second arrangement; (**c**) third arrangement.

**Figure 4:**
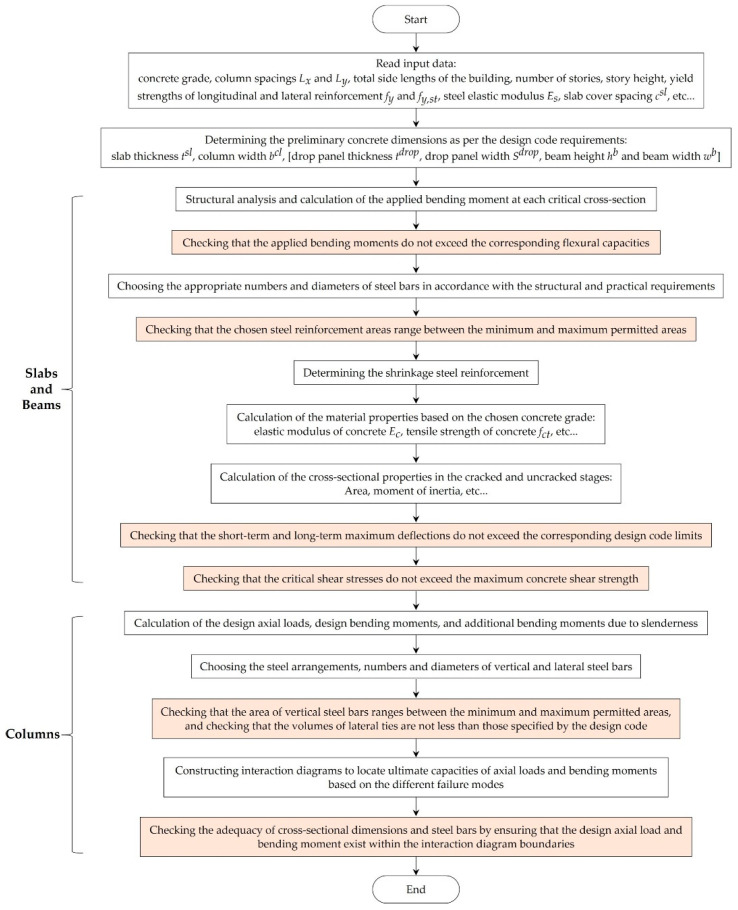
Design procedures of the structural elements for different floor systems as per ECP 203-18.

In this study, square columns were considered for simplification. These columns were classified into the interior, edge, and corner columns. Figure 3 shows the cross-sectional dimensions of columns and typical arrangements of longitudinal and transverse steel reinforcement. The axial loads and design bending moments were calculated for each column. The longitudinal and transverse steel reinforcement bars were determined to comply with the design limits. Interaction diagrams were employed to check the safety of each column. The design steps of slabs, beams, and columns are summarized in Figure 4.

## 3. Optimization Framework

### 3.1. Solver Tool

Solver, an add-in developed by frontline systems [43], is a part of Microsoft Excel’s commands suite called what-if-analysis tools. Using the solver, a user can find the optimal solution by specifying the spreadsheet cells regarding the objective function, design variables, and constraints. The objective function cell must contain a formula that directly or indirectly depends on the design variables. During the optimization process, the solver adjusts the design variable cells to fulfill the constraint cells pre-defined by the user, while all other input cells are constant. As the design variables change, the constraints and objective function are re-calculated. The main target of the solver is to find the combination of design variables that minimizes or maximizes the objective function’s cell. The solver provides three different methods to perform optimization: simplex, generalized reduced gradient (GRG), and evolutionary algorithm (EA). It is worth mentioning that the simplex and GRG methods could not deal with the design problems in the current case study due to the non-linear, non-smooth, and discontinuous nature of the objective function and design constraints.

### 3.2. Evolutionary Algorithm (EA)

The evolutionary algorithm (EA) is a meta-heuristic optimization algorithm that uses a hybrid combination of genetic algorithm and deterministic local search methods to efficiently explore the design search space. The solver begins with a random population of input values sets when EA is selected. The sets of values that produce the closest solution to the minimum or maximum target value are chosen to generate another offspring population. This process is repeated, and other populations with better characteristics are generated until one of the convergence criteria is satisfied.

### 3.3. EA Control Parameters

The solver tool enables the user to specify the control parameters from the Options tab in the solver parameters dialog box. The parameters that affect how EA converges to the best solution are discussed below.

1. Population size
  The population size refers to the number of members, (i.e., the number of different designs, each of which holds the design variables) the evolutionary method maintains simultaneously. The solver allows the user to specify a population size between 10 and 200 members. A large population size means increasing the design search space and the computational effort and, therefore, shall be related to the problem complexity.
2. Mutation rate
  The mutation rate refers to the relative frequency at which part of the population will be mutated to produce a new trial solution during each subproblem or generation. The solver allows the user to specify a mutation rate greater than 0 and less than 1%. As the mutation rate increases, the diversity of the population increases, and consequently, the probability of finding a better new solution increases.
3. Random seed
  The random seed is a positive integer number and is utilized to generate a variety of random choices. If the user operates a positive number, the evolutionary method uses the same choices each time the solver runs; otherwise, if the random seed is 0, EA may yield different solutions for different runs.
4. EA Convergence Criteria
  The solver tool enables the user to define the convergence criteria that terminate the optimization process, and these criteria are:

- Maximum difference in the objective function value
  This value refers to the maximum difference in the percentages of the objective function values for the top 99% of the generated population permitted by the solver. As this value decreases, the solution time increases, but the solver will converge at a point closer to the best solution.
- Maximum time without improvement
  This time refers to the maximum time in seconds when EA continues without a rational improvement in the objective function value of the best solution in the population. As this time elapses, the solver terminates with a message stating that the solver cannot find a better solution in the given time and reports the best solution.
- Maximum number of iterations
  This parameter specifies an upper limit for the number of iterations required for the optimization process. If the user does not set a particular number of iterations, the solver assumes that it is unlimited.

## 4. Problem Formulation

### 4.1. Design Variables

**Figure 5:**
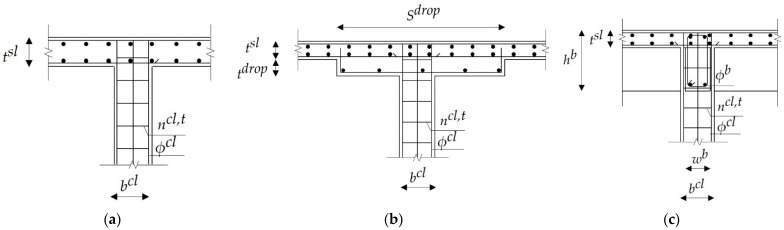
Design variables of floor systems: (**a**) FS; (**b**) FSDP; (**c**) SS.

The design variables of the optimization problem are illustrated in Table 1 and clarified in Figure 5 for each floor system. The cross-sectional dimensions of structural elements are rounded to defined increments to satisfy the construction requirements. The bar diameters are chosen from Egypt’s commercial list of steel bars.

### 4.2. Objective Function

The objective function of the design optimization problem is to minimize the building’s total cost while still fulfilling the strength and serviceability limit states of ECP 203-18 [41]. Hence, the design optimization problem can be formulated as follows:

$$\min\;f\left(x\right){=P}_{c}V_{c}{+P}_{s}W_{s}{+P}_{f}V_{c}$$

subject to

$$G_{q}^{b,\mathrm{Str}}\left(x\right)\;\leq \;1;\;q=1,\;2,\;\ldots ,\;Q$$

$$G_{r}^{sl,Str}\left(x\right)\;\leq \;1;\;r=1,\;2,\;\ldots ,\;R$$

$$G_{s}^{cl,Str}\left(x\right)\;\leq \;1;\;s=1,\;2,\;\ldots ,\;S$$

$$G_{t}^{b,Ser}\left(x\right)\;\leq \;1;\;t=1,\;2,\;\ldots ,\;T$$

$$G_{u}^{\;sl,Ser}\left(x\right)\;\leq \;1;\;u=1,\;2,\;\ldots ,\;U$$

$$G_{v}^{\;cl,Ser}\left(x\right)\;\leq \;1;\;v=1,\;2,\;\ldots ,\;V$$

(1) $$x^{lb}\;\leq \;x\;\leq {\;x}^{\mathrm{ub}}$$

where *x* is the design variables vector; f(x) is the objective function; $P_{c}$, $P_{s}$, and $P_{f}$ are the unit prices of concrete, steel, formwork, and labor, respectively; $V_{c}$ and $W_{s}$ are the total concrete volume and steel weight of the structural components, respectively; $G_{q}^{b,Str}\left(x\right)$, $G_{r}^{sl,Str}\left(x\right)$, and $G_{s}^{cl,Str}\left(x\right)$ are the strength constraint functions of beams, slabs, and columns, respectively; $G_{t}^{b,Ser}\left(x\right)$, $G_{u}^{\;sl,Ser}\left(x\right)$, and $G_{v}^{\;cl,Ser}\left(x\right)$ are the serviceability constraint functions of beams, slabs, and columns, respectively; *Q*, *R*, and *S* are the number of strength constraints regarding beams, slabs, and columns, respectively; *T*, *U*, and *V* are the number of serviceability constraints regarding beams, slabs, and columns, respectively; $x^{lb}$ and $x^{ub}$ are the lower and upper bounds of the variables vector *x*. The upper and lower bounds of each design variable are given in Table 1.

## 5. Tuning of EA Parameters

In this section, the control parameters of the evolutionary method, (i.e., population size and mutation rate) are tuned to investigate their impacts on the robustness and performance of the algorithm. The tuning was performed using fifty runs for each combination of the studied parameters. The optimization was performed on a single-story residential building with a 3 m column height. The total length in each direction of the building is 30 m, and the column spacing in each direction is 5 m. The design variables of each system are listed in Table 1. The characteristic strength $f_{cu}$ is kept constant (25 MPa) for all runs. The steel and concrete unit weights are 78.5 kN/m^3^ and 25 kN/m^3^, respectively. The yield strengths of longitudinal steel bars $f_{y}$ and lateral ties $f_{y,st}$ are 420 MPa and 240 MPa, respectively. The live load *p* and flooring load $w_{f}$ are 2 kPa and 1.5 kPa, respectively, as recommended by ECL for residential buildings. The unit prices of concrete $P_{c}$, steel $P_{s}$, and formwork and labor $P_{f}$ are 46.2 USD/m^3^, 838.8 USD/ton, and 32.4 USD/m^3^ as derived from the monthly bulletins of materials unit prices.

In this example, the default value of the maximum difference in objective function values was used (0.0001). The random seed was used to permit the generation of different solutions each time the solver ran. Therefore, the random seed value was set as zero to study the statistical performance of the evolutionary method. The maximum number of iterations was unrestricted to extend the termination duration, and the maximum time without improvement was 120 s.

### 5.1. Effects of Population Size

**Figure 6:**
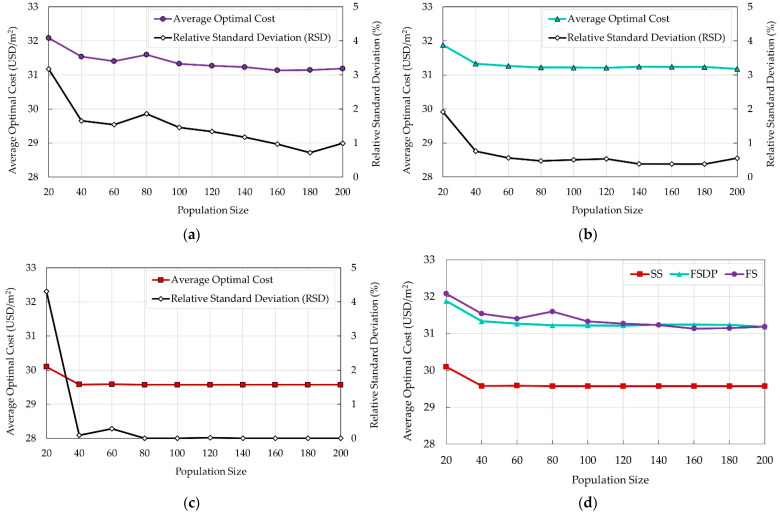
Effects of the population sizes on the average 50 runs for the optimal total costs of different floor systems: (**a**) FS; (**b**) FSDP; (**c**) SS; (**d**) all systems.

Ten values for the population size were tested (20 to 200 with an increment of 20). For each value, fifty independent runs were carried out, and the average optimal cost of the building was recorded. The relative standard deviation (RSD) was calculated for each set of runs to evaluate the variation of the randomly generated results from their average value. The default mutation rate (0.075%) was defined for all test runs. The output results are presented in Figure 6 to investigate the effects of the population sizes on the average optimal cost of each system.

It can be observed that increasing the population size improves the performance of EA in terms of the average optimal cost and RSD until the population size reaches 100 members. Further increase in the population size results in increasing the computational effort without reducing the optimal cost of the building. Additionally, the RSD tends to decrease as the population size increases. Generally, the values of RSD were small, indicating that the costs of each set of runs at the same population size are close to the average optimal cost.

### 5.2. Effects of Mutation Rate

**Figure 7:**
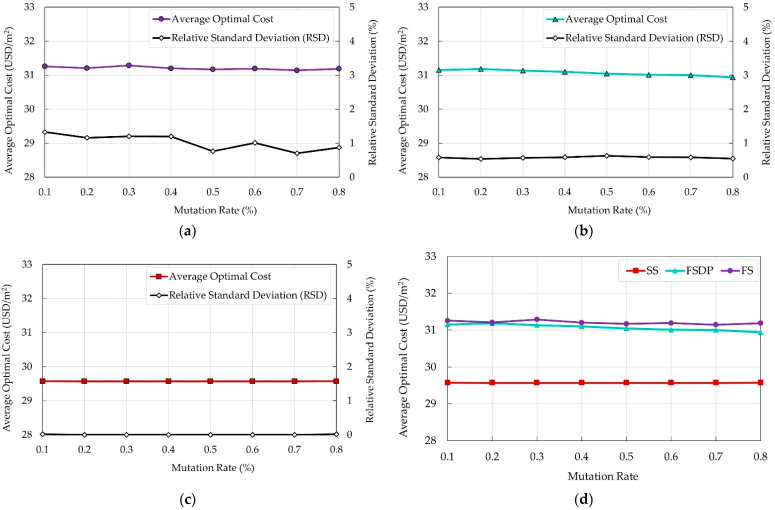
Effects of the mutation rates on the average 50 runs for the optimal total costs of different floor systems: (**a**) FS; (**b**) FSDP; (**c**) SS; (**d**) all systems.

Eight values for the mutation rate were tested (0.1 to 0.8 with increment 0.1). For each value, fifty independent runs were carried out, and the average optimal cost of the building was recorded. The RSD was also calculated for each set of runs to evaluate the deviation of the randomly generated results from their average value. The default population size (100 members) was defined for all test runs. The output results are presented in Figure 7 to investigate the effects of the mutation rates on the average optimal cost of each system.

It can be observed that increasing the mutation rate has no significant effect on the average optimal cost of the building regardless of the floor system. The RSD value was small for all floor systems. While the RSD did not exceed 1.3% and 0.7% for FS and FSDP, respectively, it was 0% for SS. Hence, the costs of each set of runs at the same population size were very close to the average optimal cost. Based on these results, the mutation rate of 0.1% is recommended to decrease the computational time of the algorithm while still obtaining good optimal results.

### 5.3. Convergence History

**Figure 8:**
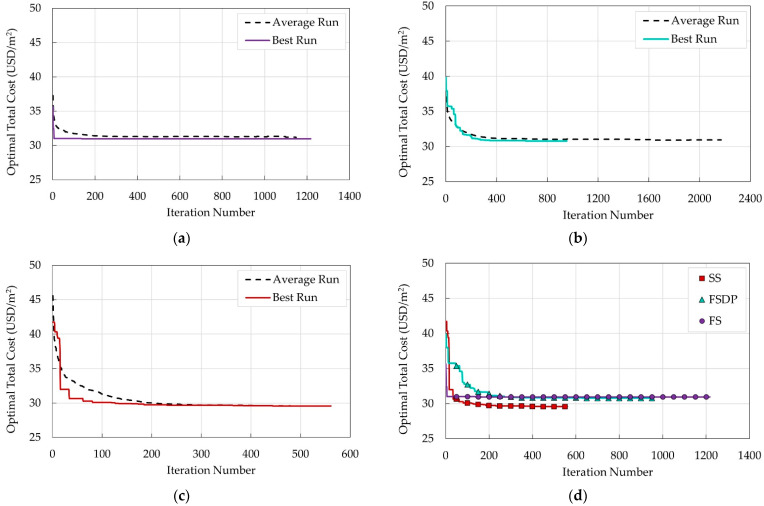
Convergence history for the average 50 runs and the best run for different floor systems: (**a**) FS; (**b**) FSDP; (**c**) SS; (**d**) all systems.

To study the convergence history, the EA parameters were kept constant, (i.e., population size = 100 members and mutation rate = 0.1%). Here, fifty runs were performed, and the convergence histories of each floor system’s average and best runs were monitored and depicted in Figure 8. Table 2 presents the optimal design results of the best run for each floor system. The optimal costs of the average and best runs for each system are recorded in Table 3. For all systems, the optimal costs of the best runs are very close to those of the average run. This means that the optimizer finds a solution near the best solution using the selected population size and mutation rate. Among all systems, SS had the least number of iterations and optimal total cost.

## 6. Case Studies and Discussion

Two case studies were employed to study the effects of the column spacings and the concrete grade on the optimal total cost of each floor system. The design variables of each floor system are listed in Table 1. The input data used in the case studies are presented in Table 4. The unit prices of materials and labor are given in Table 5.

### 6.1. Case Study 1: A Two-Story Building

**Figure 9:**
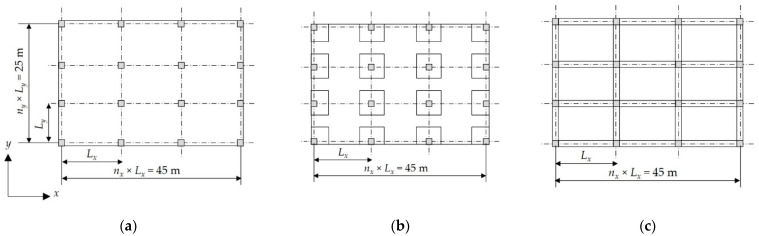
Plan layouts of the floor systems in case study 1: (**a**) FS; (**b**) FSDP; (**c**) SS.

A two-story residential building with a 3 m typical story height and a rectangular layout is considered. Figure 9 shows the plan layout of the building for each floor system. The total side lengths of the building are 45 m and 25 m in the x and y directions, respectively. In this case study, the column spacing is constant in the y-direction ($L_{y}$ = 5 m). Eight concrete grade variants, (i.e., $f_{cu}$ = 25–60 MPa) and six column spacing variants in the x-direction, (i.e., $L_{x}$ = 3.75 m, 4.09 m, 4.5 m, 5 m, 5.63 m, and 6.43 m) were considered. Due to the large set of data, the optimal design results for each floor system are presented in Tables S1–S3 in the Supplementary Materials. The population size and mutation rate values were 100 members and 0.1% during the optimization process, respectively.

#### 6.1.1. Effects of Concrete Grades

**Figure 10:**
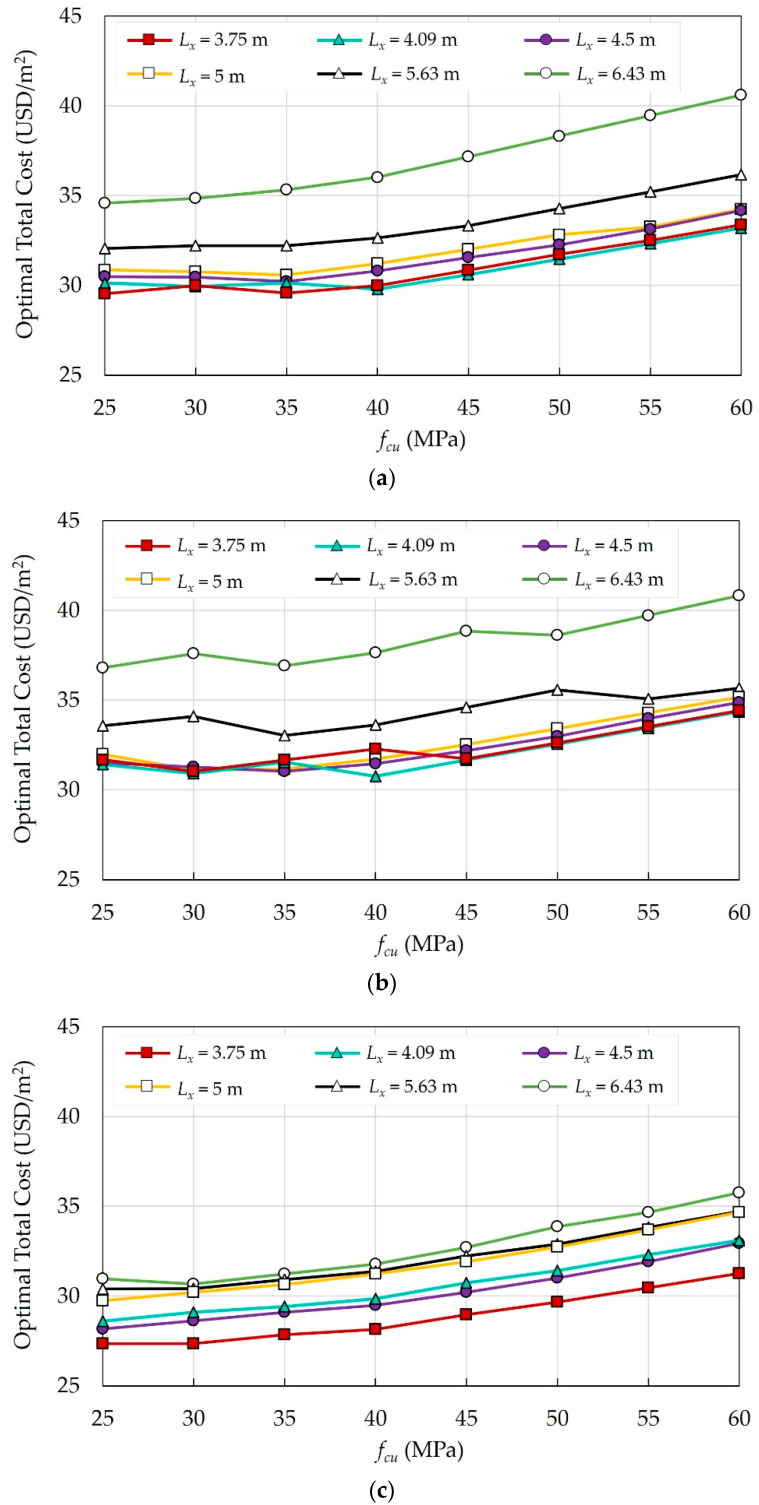
Effects of $f_{cu}$ on the optimal costs of different floor systems: (**a**) FS; (**b**) FSDP; (**c**) SS.

The effects of eight concrete grades were investigated for the current case study with different column spacings, and the optimal costs were determined. Figure 10 illustrates the impact of $f_{cu}$ on the optimal costs of each floor system. The following findings were observed:

- As $f_{cu}$ increases, $P_{c}$ increases, and consequently, the total optimal cost of the building increases. For concrete grades with $f_{cu}$ above 40 MPa, a significant increase in the total cost is observed.
- In some cases, increasing $f_{cu}$ reduces the total cost due to the substantial reduction in materials consumption. For instance, for FS at $L_{x}$ = 3.75 m, increasing $f_{cu}$ from 30 MPa to 35 MPa resulted in decreasing the slab thickness $t^{\mathrm{sl}}$ from 180 mm to 160 mm. Similarly, for SS, increasing $f_{cu}$ from 25 MPa to 30 MPa resulted in lowering the beam height $h^{b}$ and steel bar size $\phi^{cl}$ for columns and edge columns.

In the case of FSDP, a fluctuation in the total cost is observed regardless of the concrete grades. The deflection of the slabs alters the total cost significantly. As $f_{cu}$ increases, it contributes to reducing the long-term deflection, and consequently, reducing $t^{sl}$. Whenever $f_{cu}$ manages to reduce $t^{sl}$, the total cost decreases. For instance, increasing $f_{cu}$ from 35 MPa to 40 MPa resulted in reducing $t^{sl}$ from 180 mm to 160 mm. Likewise, increasing $f_{cu}$ from 30 MPa to 35 MPa resulted in reducing $t^{sl}$ from 200 mm to 180 mm.

In the case study of Ženíšek et al. [38], the results showed that for low-rise buildings, (i.e., four-story buildings) with small column spacings, (i.e., 4 m), the change in costs was insignificant as the concrete grade increased until it reached 50 MPa. Beyond this limit, the building cost significantly increases because the savings in materials quantities could not offset the high costs of high concrete grades. These results support and justify the authors’ findings.

#### 6.1.2. Effects of Column Spacing

**Figure 11:**
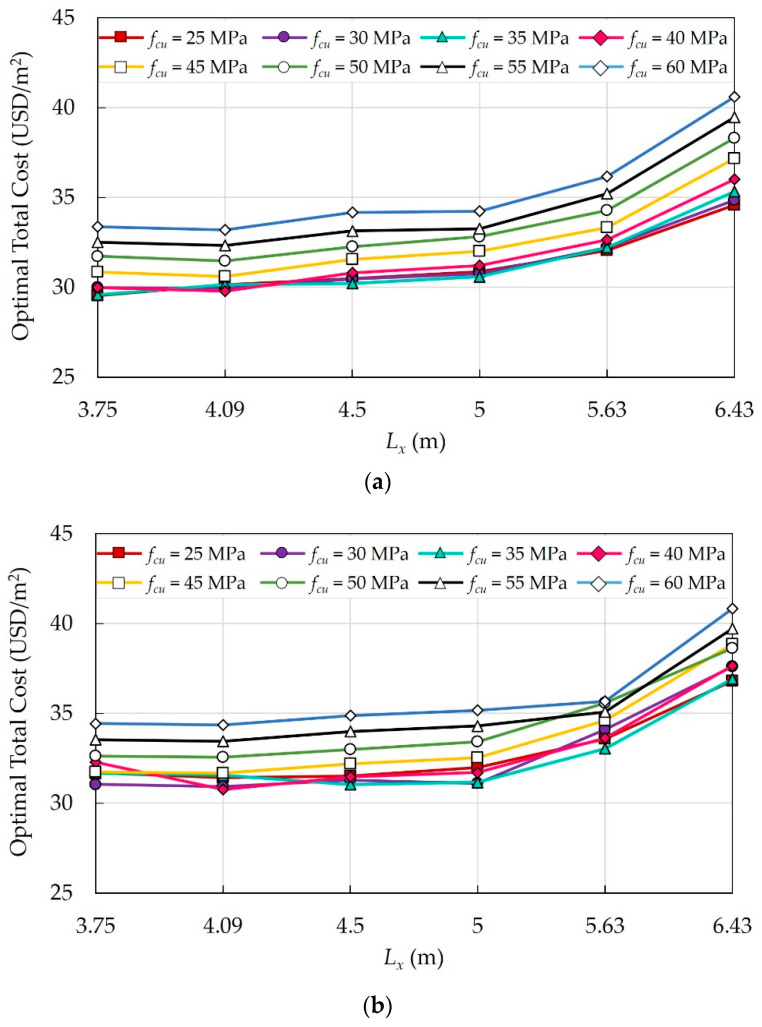

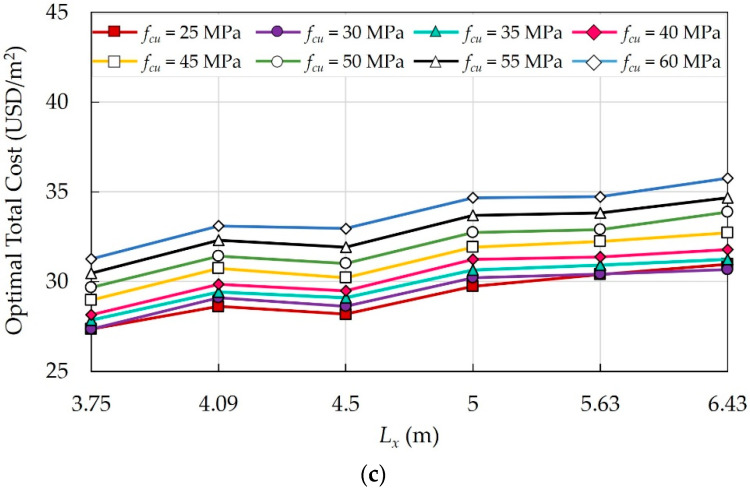
Effects of $L_{x}$ on the optimal cost of different floor systems: (**a**) FS; (**b**) FSDP; (**c**) SS.

The effects of six columns spacings in the x-direction $L_{x}$ on the optimal total costs of the building were investigated. Figure 11 illustrates the impact of $L_{x}$ on the optimal costs of different floor systems. The following findings were noted:

- For all systems, increasing $L_{x}$ usually results in increasing the total cost of the building.
- In the case of FSDP and FS, increasing $L_{x}$ up to 5 m does not yield a significant increase in the total cost because $t^{sl}$ does not increase.
- In the case of FSDP and FS, as $L_{x}$ exceeds 5 m, $t^{sl}$ increases because the deflection of flat slabs depends on the longer span, and consequently, the total cost increases significantly. In the case of SS, as $L_{x}$ exceeds 5 m, the cost variation is insignificant because the deflection of solid slabs depends on the shorter span, (i.e., $t^{sl}$ remains the same).
- In the case of SS, a significant increase in cost was observed when $L_{x}$ increased from 3.75 m to 4.09 m as $t^{sl}$ increased from 100 mm to 120 mm. Likewise, the cost increased significantly when $L_{x}$ increased from 4.5 m to 5 m as $t^{sl}$ increased from 120 mm to 140 mm. On the contrary, as $L_{x}$ increased from 4.09 m to 4.5 m, the total cost did not increase because the number of columns decreased while $t^{sl}$ remained the same.

#### 6.1.3. Comparison between Floor Systems

This section compares the total optimal costs of different floor systems at different column spacings and concrete grades. Figure S1 in the Supplementary Materials illustrates the comparison of the optimal costs for different floor systems The following findings were figured:

- In most cases, SS was the cheapest system due to the lower $t^{sl}$ and column dimensions of SS compared to FSDP and FS. This result conforms to the findings of [40].
- The variation in the total costs of all systems is insignificant at $L_{x}$ = 4.09 m and 5 m.
- As $L_{x}$ exceeds 5 m, the deflection of SS remains constant, while the deflection of FS and FSDP increases significantly. Therefore, the maximum cost saving between SS and other systems can be observed at $L_{x}$ = 6.43 m.

### 6.2. Case Study 2: A Four-Story Building

**Figure 12:**
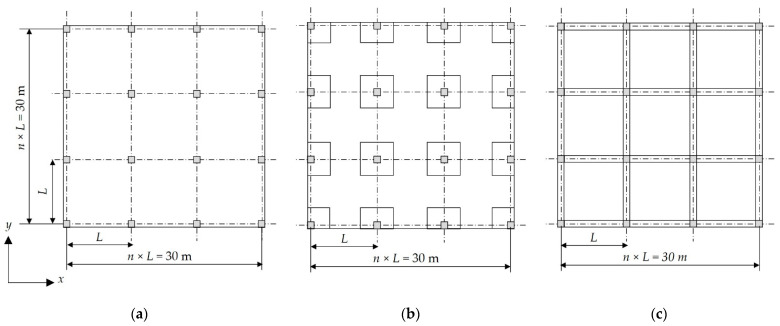
Plan layouts of the floor systems in case study 2: (**a**) FS; (**b**) FSDP; (**c**) SS.

A four-story residential building with a 3.3 m typical story height and a square layout was considered. The total side length of the building in each direction is 30 m. In this case study, eight concrete grade variants, (i.e., $f_{cu}$ = 25–60 MPa) and six column spacing variants (*L* = 3.3 m, 3.75 m, 4.29 m, 5 m, 6 m, 7.5 m) were considered to investigate the effects of concrete grades and column spacings on the optimal total cost of the building. Here, the column spacings are simultaneously adjusted in the x and y directions. Figure 12 shows the plan layout of the building for each floor system. Due to the large set of data, the optimal design results for each floor system are presented in Tables S4–S6 in the Supplementary Materials. The population size was 100 members, and the mutation rate was 0.1% during the optimization process.

#### 6.2.1. Effects of Concrete Grades

**Figure 13:**
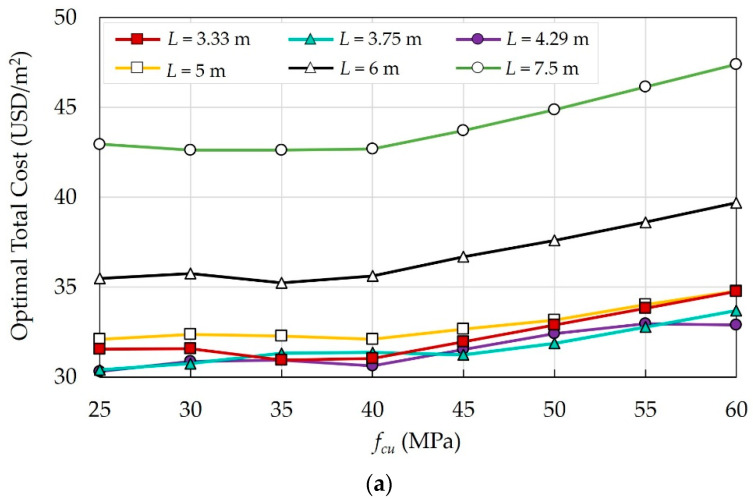

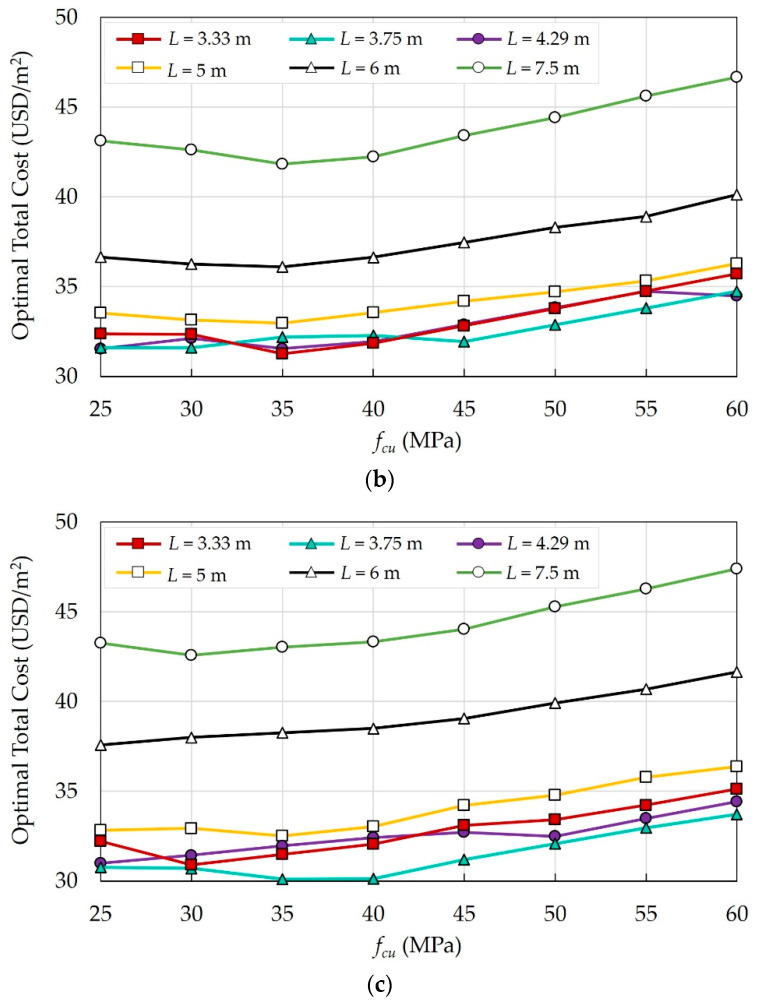
Effects of $f_{cu}$ on the optimal costs of different floor systems: (**a**) FS; (**b**) FSDP; (**c**) SS.

The effects of eight concrete grades were investigated for the building under consideration, and the optimal costs were determined. Figure 13 illustrates the impact of $f_{cu}$ on the optimal costs of different floor systems.

For all systems, increasing $f_{cu}$ increases the building’s total cost. However, in some cases, increasing $f_{cu}$ enhances the mechanical properties, reduces the cross-sectional dimensions of the structural elements, and consequently reduces the total cost of the building. As $f_{cu}$ exceeds 40 MPa, the increase in cost becomes significant.

#### 6.2.2. Effects of Column Spacing

**Figure 14:**
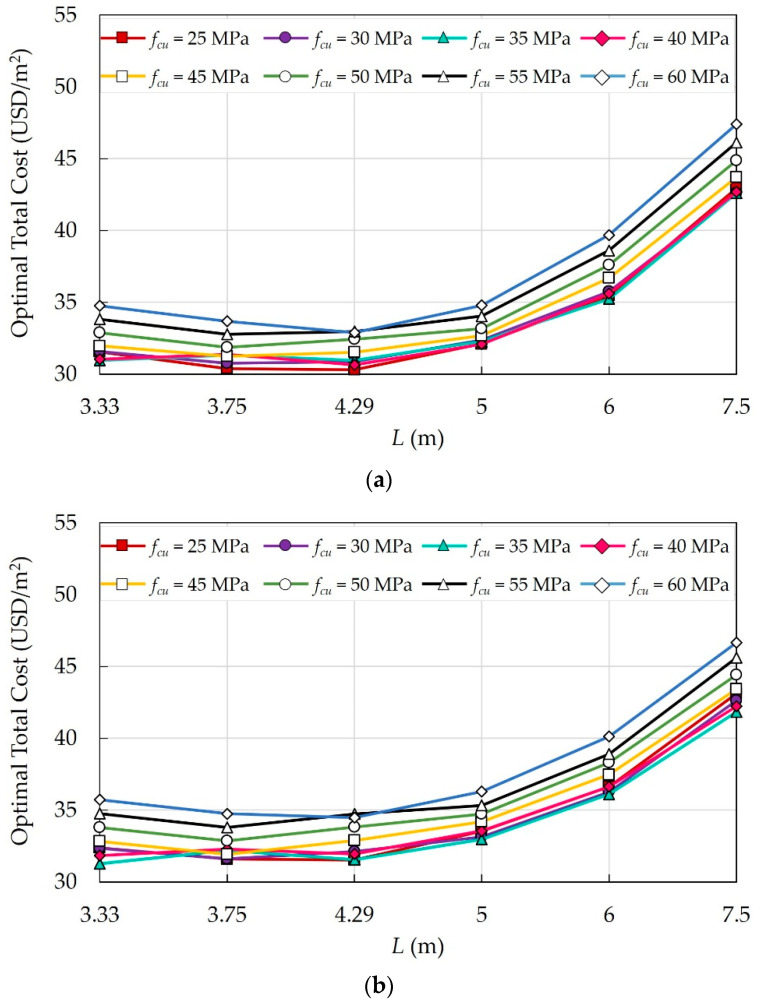

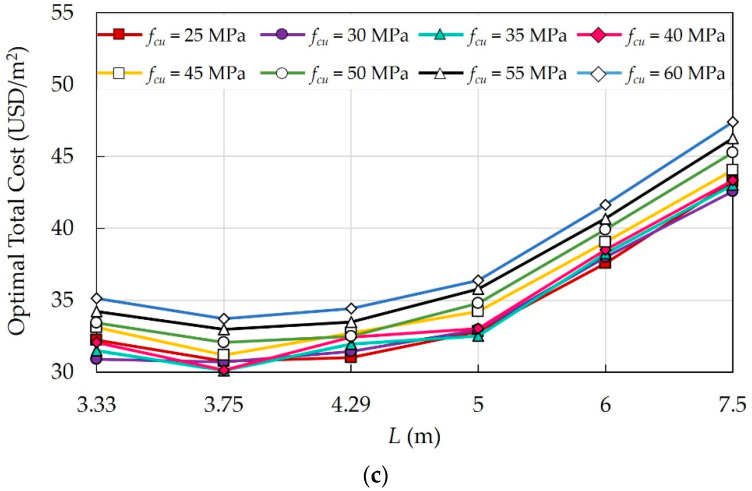
Effects of *L* on the optimal costs of different floor systems: (**a**) FS; (**b**) FSDP; (**c**) SS.

The effects of six columns spacings *L* in both directions on the optimal total costs of the building were investigated. Figure 14 shows the impact of *L* on the optimal costs of different floor systems.

For all floor systems, as *L* exceeds 5 m, the deflection of slabs increases, and consequently, the total cost increases significantly. As *L* increased from 3.3 m to 3.75 m, the total cost decreased because the number of columns decreased while $t^{sl}$ remained the same.

#### 6.2.3. Comparison between Floor Systems

To determine the most economical system, the total optimal costs of different floor systems at different column spacings and concrete grades were compared. Figure S2 in the Supplementary Materials illustrates the comparison of the optimal costs for different floor systems. The following findings were observed:

- In this case study, the slenderness effect increased the optimal cross-sectional dimensions of columns of SS, and FS produced the highest cost savings. In a similar case study [40], SS was the cheapest among the floor systems; however, the additional bending moment due to column slenderness was not considered. Thus, more investigation is needed to confirm the impact of the column height on the choice of the best floor system.
- In the case of SS, as *L* exceeded 5 m, $t^{sl}$ increased, and additional steel reinforcement meshes were installed to resist the shrinkage effects imposed by the design code requirements. Therefore, SS has become the most expensive system.
- At *L* = 7.5 m, the costs of all systems are almost the same.

## 7. Conclusions

In this paper, a cost optimization model was utilized to investigate the effects of the column spacings and concrete grades on the optimal costs of RC buildings. The model was developed using Microsoft Excel because most designers in the design office practice are familiar with the spreadsheets. The evolutionary algorithm (EA) available in Microsoft Excel’s solver add-in was utilized to optimize the buildings. Three floor systems were considered: FS, FSDP, and SS. For each system, the serviceability and ultimate limit states were checked based on the design regulations of ECP 203-18. The design variables were the cross-sectional dimensions and steel bars of different structural elements. The sizes of steel bars were selected from a commercial database derived from steel factories in Egypt to consider the practical requirements.

The EA control parameters were tuned to evaluate the effects of these parameters on the optimal costs and computational time. The results of the tuning showed that:

- For all buildings investigated, a population size of 100 members was adequate to minimize the cost and the computational effort.
- Increasing the mutation rate up to 0.8% had no significant effect on the optimal costs, and hence, a mutation rate of 0.1% was sufficient to obtain good results.
- Designers may run the solver several times to obtain a better solution.

Two case studies were considered to examine the effects of the column spacings and concrete grades on the optimal costs of each floor system. The following conclusions were drawn:

- For lower concrete grades ($f_{cu}$ up to 40 MPa), the change in total costs of all floor systems is unpredictable as $f_{cu}$ changes.
- The total cost of the building is affected majorly by the slab thickness. If increasing $f_{cu}$ manages to reduce the slab thickness, the total cost decreases; otherwise, the total cost increases due to the unit price of the higher concrete grade.
- For concrete grades with $f_{cu}$ above 40 MPa, the costs increase significantly due to the high unit prices of these grades. The cost reduction resulting from quantities savings was insufficient to offset the high unit costs of concrete grades above 40 MPa.
- For two-story buildings with 3 m high columns, SS was the cheapest floor system. For four-story buildings with 3.3 m high columns, FS was the most affordable system. Hence, the column height may affect the choice of the optimal floor system.
- For all systems, increasing the column spacings in both directions above 5 m increases the total cost significantly due to the high deflection.

The results obtained from the current study provide guidelines for the designers to select the most suitable column spacings and concrete grades for low-rise RC residential buildings. The economic parameters in the current study were limited to the direct construction costs of materials and labor. Other aspects could be included in the objective function to account for the overall expenditure such as the operational, maintenance, and repair costs during the life span of the buildings. The environmental impacts of the construction materials could also be considered to achieve a sustainable optimal design. The presented methodology could be applied to buildings with different functions, (i.e., office buildings, hospitals, etc.), to consider the various magnitudes of live loads and column heights on the optimal costs.

## Figures and Tables

**Table 1:** Design variables of each floor system.

	Design Variable	Number of Variables	Variable Range	Step Size	FS	FSDP	SS
$t^{sl}$	Slab thickness (mm)	1	150–300 for FSDP and FS 80–300 for SS	20	*✓*	*✓*	*✓*
$h^{b}$	Beam thickness (mm)	1	400–900	50	-	-	*✓*
$w^{b}$	Beam width (mm)	1	250–400	50	-	-	*✓*
$\phi^{b}$	Beam bar size (mm)	1	10, 12, 16, 18, 22, and 25	-	-	-	*✓*
$t^{drop}$	Drop panel thickness (mm)	1	40–120	20	-	*✓*	-
$S^{drop}$	Drop panel width (mm)	1	1500–2500	50	-	*✓*	-
$b^{cl}$	Column width (mm)	4	300–800 for FS and FSDP 250–800 for SS	50	*✓*	*✓*	*✓*
$\phi^{cl}$	Column bar size (mm)	4	12, 16, 18, 22, 25, and 28	-	*✓*	*✓*	*✓*
$n^{cl}$	Number of column lateral ties	4	5–10	1	*✓*	*✓*	*✓*

**Table 2:** Summary of the optimal design results of the best run for each floor system.

Floor System	Floor					Interior Columns		Edge Columns (x-Direction)		Edge Columns (y-Direction)		Corner Columns	
	$t^{\mathrm{sl}}$ (mm)	$t^{\mathrm{drop}}$ (mm)	$S^{\mathrm{drop}}$ (mm)	$h^{b}$ (mm)	$w^{b}$ (mm)	$b^{\mathrm{cl}}$ (mm)	Steel Bars	$b^{\mathrm{cl}}$ (mm)	Steel Bars	$b^{\mathrm{cl}}$ (mm)	Steel Bars	$b^{\mathrm{cl}}$ (mm)	Steel Bars
FS	160	-	-	-	-	500	8T18	400	8T16	400	8T16	350	8T16
FSDP	160	100	1800	-	-	300	4T16	350	8T16	300	4T22	350	8T16
SS	140	-	-	500	250	250	4T16	250	4T16	250	4T16	250	4T16

**Table 3:** Optimal total costs recorded from the convergence history of each floor system.

Floor System	Run Type	Optimal Cost (USD/m^2^)
FS	Average	31.24
	Best	30.96
FSDP	Average	31.09
	Best	30.79
SS	Average	29.78
	Best	29.57

**Table 4:** Input data of the optimization design model.

Parameter	Value
Yield strength of the high tensile steel (longitudinal bars)	420 MPa
Yield strength of the mild steel (lateral bars)	240 MPa
Steel’s elastic modulus	200 GPa
Concrete’s unit weight	25 kN/m^3^
Steel’s unit weight	78.5 kN/m^3^
Brick’s unit weight (partition walls)	14 kN/m^3^
Concrete cover spacing for slabs	25 mm
Concrete cover spacing for beams	50 mm
Concrete cover spacing for columns	25 mm
Live load	2 kN/m^2^
Flooring load	1.5 kN/m^2^
Bar diameter of lateral ties	8 mm

**Table 5:** Materials and labor unit prices.

	Component	Strength (MPa)	Price (USD/Unit)	Unit
$P_{c}$	Concrete	25	46.2	m^3^
		30	49.2	
		35	52.2	
		40	55.1	
		45	60.0	
		50	64.9	
		55	69.7	
		60	74.6	
$P_{s}$	High tensile steel	420	837.8	ton
	Mild steel	240	837.8	
$P_{f}$	Formwork and labor	-	32.4	m^3^

## Data Availability

All data generated or used during the study are available from the corresponding author by request.

## Supplementary Materials

The following supporting information can be downloaded at: https://www.mdpi.com/article/10.3390/ma15124290/s1, Figure S1. Effects of "L" _"x" on the optimal costs of different floor systems in case study 1: (a) "f" _"cu" = 25 MPa; (b) "f" _"cu" = 30 MPa; (c) "f" _"cu" = 35 MPa; (d) "f" _"cu" = 40 MPa; (e) "f" _"cu" = 45 MPa; (f) "f" _"cu" = 50 MPa; (g) "f" _"cu" = 55 MPa; (h) "f" _"cu" = 60 MPa. Figure S2. Effects of the L on the optimal costs of different floor systems in case study 2: (a) "f" _"cu" = 25 MPa; (b) "f" _"cu" = 30 MPa; (c) "f" _"cu" = 35 MPa; (d) "f" _"cu" = 40 MPa; (e) "f" _"cu" = 45 MPa; (f) "f" _"cu" = 50 MPa; (g) "f" _"cu" = 55 MPa; (h) "f" _"cu" = 60 MPa. Table S1. Summary of FS optimal results for different column spacing and concrete grade variants (case study 1). Table S2. Summary of FSDP optimal results for different column spacing and concrete grade variants (case study 1). Table S3. Summary of SS optimal results for different column spacing and concrete grade variants (case study 1). Table S4. Summary of FS optimal results for different column spacing and concrete grade variants (case study 2). Table S5. Summary of FSDP optimal results for different column spacing and concrete grade variants (case study 2). Table S6. Summary of SS optimal results for different column spacing and concrete grade variants (case study 2).
