# Supplementary material for: Effects of Concrete Grades and Column Spacings on the Optimal Design of Reinforced Concrete Buildings
Source: Materials (Basel). 2022 Jun 17;15(12):4290. doi: 10.3390/ma15124290 (PMC9228045; doi:10.3390/ma15124290)
Supplement: Supplementary file 1 [file materials-15-04290-s001.zip › materials-1758243-supplementary.pdf]

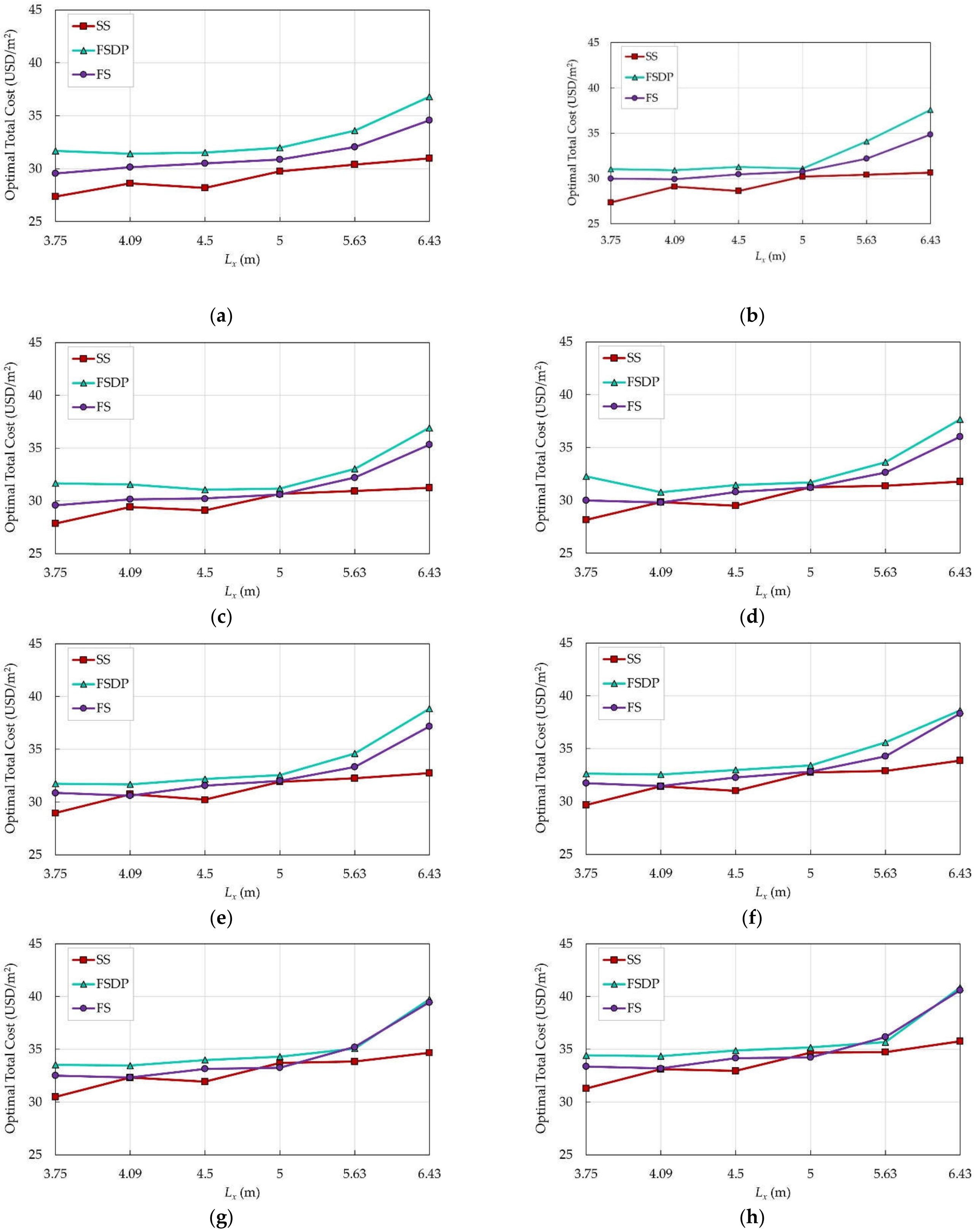

Figure S1. Effects of L_x on the optimal costs of different floor systems in case study 1: (**a**) f_{cu} = 25 MPa; (**b**) f_{cu} = 30 MPa; (**c**) f_{cu} = 35 MPa; (**d**) f_{cu} = 40 MPa; (**e**) f_{cu} = 45 MPa; (**f**) f_{cu} = 50 MPa; (**g**) f_{cu} = 55 MPa; (**h**) f_{cu} = 60 MPa.

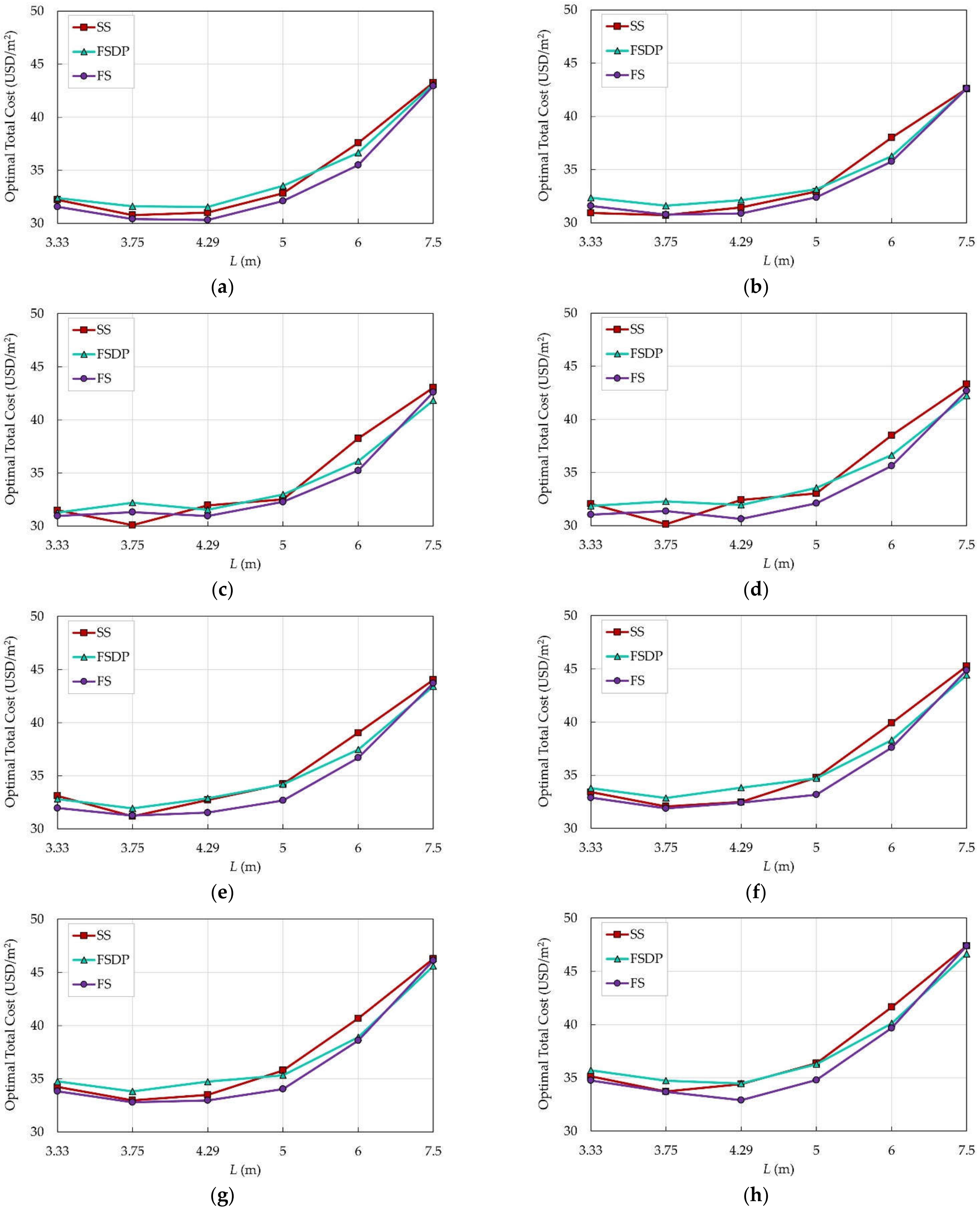

Figure S2. Effects of the L on the optimal costs of different floor systems in case study 2: (**a**) f_{cu} = 25 MPa; (**b**) f_{cu} = 30 MPa; (**c**) f_{cu} = 35 MPa; (**d**) f_{cu} = 40 MPa; (**e**) f_{cu} = 45 MPa; (**f**) f_{cu} = 50 MPa; (**g**) f_{cu} = 55 MPa; (**h**) f_{cu} = 60 MPa.

Table S1. Summary of FS optimal results for different column spacing and concrete grade variants (case study 1).

f_{cu} (MPa)	$n_x \times L_x$ (mm)	Floors	Interior Columns			Edge Columns (x-direction)			Edge Columns (y-direction)			Corner Columns			Total Cost (USD/m^2)
		t^{sl} (mm)	b^{cl} (mm)	Steel bars	No. of Columns	b^{cl} (mm)	Steel bars	No. of Columns	b^{cl} (mm)	Steel bars	No. of Columns	b^{cl} (mm)	Steel bars	No. of Columns	
25	7 × 6429	220	450	8T18	24	350	8T16	12	400	8T16	8	400	8T16	4	34.57
25	8 × 5625	180	500	8T18	28	400	8T16	14	400	8T16	8	350	8T16	4	32.06
25	9 × 5000	160	500	8T18	32	400	8T16	16	400	8T16	8	350	8T16	4	30.87
25	10 × 4500	160	450	8T18	36	350	8T16	8	350	8T16	18	350	8T16	4	30.49
25	11 × 4091	160	400	8T16	40	350	8T16	8	350	8T16	20	300	4T16	4	30.14
25	12 × 3750	180	300	4T16	44	350	8T16	8	300	4T16	22	300	4T16	4	29.55
30	7 × 6429	220	400	8T16	24	350	8T16	12	400	8T16	8	400	8T16	4	34.85
30	8 × 5625	180	450	8T18	28	350	8T16	14	400	8T16	8	350	8T16	4	32.21
30	9 × 5000	180	400	8T16	32	300	4T18	16	300	4T18	8	300	4T22	4	30.77
30	10 × 4500	160	400	8T16	36	350	8T16	8	350	8T16	18	300	4T18	4	30.46
30	11 × 4091	180	300	4T16	40	300	4T18	8	300	4T16	20	300	4T18	4	29.94
30	12 × 3750	180	300	4T16	44	300	4T18	8	300	4T16	22	300	4T16	4	29.99
35	7 × 6429	220	350	8T16	24	350	8T16	12	400	8T16	8	400	8T16	4	35.32
35	8 × 5625	180	400	8T16	28	350	8T16	14	350	8T16	8	350	8T16	4	32.21
35	9 × 5000	160	400	8T16	32	350	8T16	16	350	8T16	8	300	4T18	4	30.59
35	10 × 4500	180	300	4T16	36	300	4T18	8	300	4T18	18	300	4T18	4	30.22
35	11 × 4091	160	350	8T16	40	300	4T18	8	300	4T16	20	300	4T16	4	30.14
35	12 × 3750	160	300	4T16	44	300	4T18	8	300	4T16	22	300	4T16	4	29.58
40	7 × 6429	220	350	8T16	24	350	8T16	12	400	8T16	8	400	8T16	4	36.02
40	8 × 5625	180	400	8T16	28	300	4T22	14	350	8T16	8	350	8T16	4	32.64
40	9 × 5000	160	400	8T16	32	350	8T16	16	350	8T16	8	350	8T16	4	31.22
40	10 × 4500	180	300	4T16	36	300	4T18	8	300	4T18	18	300	4T18	4	30.80
40	11 × 4091	160	300	4T16	40	300	4T18	8	300	4T16	20	300	4T18	4	29.80
40	12 × 3750	160	300	4T16	44	300	4T18	8	300	4T16	22	300	4T16	4	30.00

f_{cu} (MPa)	$n_x \times L_x$ (mm)	Floors	Interior Columns			Edge Columns (*x*-direction)			Edge Columns (*y*-direction)			Corner Columns			Total Cost (USD/m²)
		t^{sl} (mm)	b^{cl} (mm)	Steel bars	No. of Columns	b^{cl} (mm)	Steel bars	No. of Columns	b^{cl} (mm)	Steel bars	No. of Columns	b^{cl} (mm)	Steel bars	No. of Columns	
45	7 × 6429	220	350	8T16	24	350	8T16	12	400	8T16	8	400	8T16	4	37.17
45	8 × 5625	180	350	8T16	28	300	4T22	14	300	4T22	8	300	4T22	4	33.33
45	9 × 5000	160	400	8T16	32	350	8T16	16	350	8T16	8	300	4T18	4	32.01
45	10 × 4500	160	350	8T16	36	350	8T16	8	300	4T16	18	300	4T18	4	31.55
45	11 × 4091	160	300	4T16	40	300	4T18	8	300	4T16	20	300	4T18	4	30.60
45	12 × 3750	160	300	4T16	44	300	4T18	8	300	4T16	22	300	4T16	4	30.86
50	7 × 6429	220	350	8T16	24	350	8T16	12	400	8T16	8	400	8T16	4	38.31
50	8 × 5625	180	350	8T16	28	300	4T22	14	300	4T22	8	300	4T22	4	34.27
50	9 × 5000	160	400	8T16	32	350	8T16	16	350	8T16	8	300	4T18	4	32.82
50	10 × 4500	160	350	8T16	36	300	4T18	8	300	4T16	18	300	4T18	4	32.27
50	11 × 4091	160	300	4T16	40	300	4T18	8	300	4T16	20	300	4T18	4	31.46
50	12 × 3750	160	300	4T16	44	300	4T18	8	300	4T16	22	300	4T16	4	31.72
55	7 × 6429	220	350	8T16	24	350	8T16	12	400	8T16	8	400	8T16	4	39.46
55	8 × 5625	180	350	8T16	28	300	4T22	14	300	4T22	8	300	4T22	4	35.22
55	9 × 5000	160	400	8T16	32	300	4T18	16	300	4T18	8	350	8T16	4	33.26
55	10 × 4500	160	350	8T16	36	300	4T18	8	300	4T16	18	300	4T18	4	33.13
55	11 × 4091	160	300	4T16	40	300	4T18	8	300	4T16	20	300	4T18	4	32.34
55	12 × 3750	160	300	4T16	44	300	4T18	8	300	4T16	22	300	4T16	4	32.50
60	7 × 6429	220	350	8T16	24	350	8T16	12	350	8T18	8	350	8T18	4	40.60
60	8 × 5625	180	350	8T16	28	300	4T22	14	300	4T22	8	300	4T22	4	36.17
60	9 × 5000	160	400	8T16	32	300	4T18	16	350	8T16	8	300	4T18	4	34.23
60	10 × 4500	160	350	8T16	36	350	8T16	8	300	4T16	18	300	4T18	4	34.16
60	11 × 4091	160	300	4T16	40	300	4T18	8	300	4T16	20	300	4T18	4	33.19
60	12 × 3750	160	300	4T16	44	300	4T18	8	300	4T16	22	300	4T16	4	33.37

Table S2. Summary of FSDP optimal results for different column spacing and concrete grade variants (case study 1).

f_{cu} (MPa)	$n_x \times L_x$ (mm)	Floors			Interior Columns			Edge Columns (x-direction)			Edge Columns (y-direction)			Corner Columns			Total Cost (USD/m²)
		t^{sl} (mm)	t^{drop} (mm)	S^{drop} (mm)	b^{cl} (mm)	Steel bars	No. of Columns	b^{cl} (mm)	Steel bars	No. of Columns	b^{cl} (mm)	Steel bars	No. of Columns	b^{cl} (mm)	Steel bars	No. of Columns	
25	7 × 6429	240	60	2200	350	8T16	24	350	8T16	12	400	8T22	8	400	8T16	4	37.12
25	8 × 5625	200	60	2500	400	8T16	28	350	8T16	14	350	8T22	8	300	4T16	4	33.82
25	9 × 5000	180	60	2500	350	8T16	32	350	8T16	16	350	8T22	8	350	8T16	4	32.30
25	10 × 4500	180	60	2000	350	8T16	36	350	8T16	8	300	4T22	18	350	8T16	4	31.87
25	11 × 4091	180	60	2000	400	8T16	40	300	4T16	8	300	4T22	20	300	4T16	4	31.81
25	12 × 3750	200	60	1800	300	4T16	44	300	4T18	8	300	4T22	22	300	4T16	4	32.12
30	7 × 6429	240	60	2200	350	8T16	24	350	8T16	12	400	8T22	8	400	8T16	4	37.91
30	8 × 5625	200	60	2400	300	4T22	28	300	4T22	14	350	8T22	8	300	4T16	4	34.33
30	9 × 5000	160	40	2000	300	4T16	32	300	4T18	16	500	8T22	8	350	8T16	4	31.34
30	10 × 4500	180	60	2000	300	4T16	36	300	4T18	8	300	4T22	18	300	4T16	4	31.51
30	11 × 4091	180	60	2000	300	4T16	40	300	4T18	8	300	4T22	20	300	4T16	4	31.30
30	12 × 3750	180	60	1800	300	4T16	44	300	4T18	8	300	4T22	22	300	4T16	4	31.48
35	7 × 6429	220	60	2200	350	8T16	24	350	8T16	12	400	8T22	8	350	8T16	4	37.20
35	8 × 5625	180	60	2000	300	4T16	28	350	8T16	14	300	4T22	8	300	4T16	4	32.96
35	9 × 5000	160	40	2450	300	4T16	32	300	4T22	16	350	8T22	8	300	4T16	4	31.39
35	10 × 4500	160	40	1850	300	4T16	36	400	8T16	8	300	4T22	18	300	4T16	4	31.38
35	11 × 4091	180	60	2000	300	4T16	40	300	4T18	8	300	4T22	20	300	4T16	4	31.92
35	12 × 3750	180	60	1800	300	4T16	44	300	4T18	8	300	4T22	22	300	4T16	4	32.10
40	7 × 6429	220	60	2200	350	8T16	24	350	8T16	12	400	8T22	8	350	8T16	4	37.92
40	8 × 5625	180	60	2000	300	4T16	28	350	8T16	14	300	4T22	8	300	4T16	4	33.54
40	9 × 5000	160	40	2450	300	4T16	32	300	4T22	16	350	8T22	8	300	4T16	4	31.94
40	10 × 4500	160	60	2000	300	4T16	36	350	8T16	8	300	4T22	18	350	8T16	4	31.81
40	11 × 4091	160	40	2000	300	4T16	40	300	4T18	8	300	4T22	20	300	4T16	4	31.14
40	12 × 3750	180	60	1800	300	4T16	44	300	4T18	8	300	4T22	22	300	4T16	4	32.72

f_{cu} (MPa)	$n_x \times L_x$ (mm)	Floors			Interior Columns			Edge Columns (x-direction)			Edge Columns (y-direction)			Corner Columns			Total Cost (USD/m²)
		t^{sl} (mm)	t^{drop} (mm)	S^{drop} (mm)	b^{cl} (mm)	Steel bars	No. of Columns	b^{cl} (mm)	Steel bars	No. of Columns	b^{cl} (mm)	Steel bars	No. of Columns	b^{cl} (mm)	Steel bars	No. of Columns	
45	7 × 6429	220	60	2200	350	8T16	24	350	8T16	12	400	8T22	8	350	8T16	4	39.11
45	8 × 5625	180	60	2000	300	4T16	28	350	8T16	14	300	4T22	8	300	4T16	4	34.52
45	9 × 5000	160	40	2000	300	4T16	32	300	4T22	16	350	8T22	8	300	4T16	4	32.77
45	10 × 4500	160	40	2100	300	4T16	36	350	8T16	8	300	4T22	18	400	8T16	4	32.55
45	11 × 4091	160	40	2000	300	4T16	40	300	4T18	8	300	4T22	20	300	4T16	4	32.03
45	12 × 3750	160	40	1800	300	4T16	44	300	4T18	8	300	4T22	22	300	4T16	4	32.17
50	7 × 6429	200	60	2500	350	8T16	24	350	8T16	12	400	8T22	8	400	8T16	4	38.94
50	8 × 5625	180	60	2000	300	4T16	28	350	8T16	14	300	4T22	8	300	4T16	4	35.50
50	9 × 5000	160	40	2000	300	4T16	32	300	4T22	16	350	8T22	8	300	4T16	4	33.65
50	10 × 4500	160	40	2250	300	4T16	36	350	8T16	8	300	4T22	18	350	8T16	4	33.24
50	11 × 4091	160	40	2000	300	4T16	40	300	4T18	8	300	4T22	20	300	4T16	4	32.93
50	12 × 3750	160	40	1800	300	4T16	44	300	4T18	8	300	4T22	22	300	4T16	4	33.07
55	7 × 6429	200	60	2500	350	8T16	24	350	8T16	12	350	8T22	8	400	8T16	4	39.95
55	8 × 5625	160	40	2000	300	4T16	28	300	4T22	14	300	4T22	8	300	4T16	4	35.00
55	9 × 5000	160	40	2000	300	4T16	32	300	4T22	16	350	8T22	8	300	4T16	4	34.53
55	10 × 4500	160	40	2000	300	4T16	36	350	8T16	8	300	4T22	18	400	8T16	4	34.34
55	11 × 4091	160	40	2000	300	4T16	40	300	4T18	8	300	4T22	20	300	4T16	4	33.83
55	12 × 3750	160	40	1800	300	4T16	44	300	4T18	8	300	4T22	22	300	4T16	4	33.97
60	7 × 6429	200	60	2500	350	8T16	24	350	8T16	12	400	8T22	8	400	8T16	4	41.15
60	8 × 5625	160	40	2500	300	4T16	28	300	4T22	14	300	4T22	8	300	4T16	4	35.59
60	9 × 5000	160	40	2000	300	4T16	32	300	4T22	16	350	8T22	8	300	4T16	4	35.41
60	10 × 4500	160	40	2000	300	4T16	36	350	8T16	8	300	4T22	18	400	8T16	4	35.23
60	11 × 4091	160	40	2000	300	4T16	40	300	4T18	8	300	4T22	20	300	4T16	4	34.72
60	12 × 3750	160	40	1800	300	4T16	44	300	4T18	8	300	4T22	22	300	4T16	4	34.86

Table S3. Summary of SS optimal results for different column spacing and concrete grade variants (case study 1).

f_{cu} (MPa)	$n_x \times L_x$ (mm)	Floors			Interior Columns			Edge Columns (x-direction)			Edge Columns (y-direction)			Corner Columns			Total Cost (USD/m²)
		t^{sl} (mm)	h^b (mm)	w^b (mm)	b^{cl} (mm)	Steel bars	No. of Columns	b^{cl} (mm)	Steel bars	No. of Columns	b^{cl} (mm)	Steel bars	No. of Columns	b^{cl} (mm)	Steel bars	No. of Columns	
25	7 × 6429	140	650	250	300	4T22	24	300	4T16	12	250	4T16	8	250	4T16	4	30.74
25	8 × 5625	140	550	250	300	4T18	28	350	8T16	14	250	4T16	8	250	4T16	4	30.39
25	9 × 5000	140	500	250	300	4T16	32	300	4T16	16	250	4T16	8	250	4T16	4	29.75
25	10 × 4500	120	450	250	300	4T16	36	300	4T16	8	250	4T16	18	250	4T16	4	28.19
25	11 × 4091	120	450	250	300	4T16	40	300	4T16	8	250	4T16	20	250	4T16	4	28.62
25	12 × 3750	100	450	250	250	4T18	44	300	4T16	8	250	4T16	22	250	4T16	4	27.36
30	7 × 6429	140	600	250	300	4T16	24	300	4T16	12	250	4T16	8	250	4T16	4	30.67
30	8 × 5625	140	500	250	300	4T16	28	300	4T16	14	250	4T16	8	250	4T16	4	30.43
30	9 × 5000	140	500	250	300	4T16	32	300	4T16	16	250	4T16	8	250	4T16	4	30.21
30	10 × 4500	120	450	250	300	4T16	36	300	4T16	8	250	4T16	18	250	4T16	4	28.63
30	11 × 4091	120	450	250	300	4T16	40	300	4T16	8	250	4T16	20	250	4T16	4	29.10
30	12 × 3750	100	400	250	250	4T16	44	250	4T16	8	250	4T16	22	250	4T16	4	27.36
35	7 × 6429	140	550	250	300	4T16	24	300	4T16	12	250	4T16	8	250	4T16	4	31.24
35	8 × 5625	140	450	250	300	4T16	28	300	4T16	14	250	4T16	8	250	4T16	4	30.93
35	9 × 5000	140	450	250	300	4T16	32	300	4T16	16	250	4T16	8	250	4T16	4	30.65
35	10 × 4500	120	450	250	250	4T16	36	300	4T16	8	300	4T16	18	250	4T16	4	29.10
35	11 × 4091	120	400	250	250	4T16	40	250	4T16	8	250	4T16	20	250	4T16	4	29.42
35	12 × 3750	100	400	250	250	4T16	44	250	4T16	8	250	4T16	22	250	4T16	4	27.85
40	7 × 6429	140	500	250	300	4T16	24	300	4T16	12	250	4T16	8	250	4T16	4	31.78
40	8 × 5625	140	450	250	300	4T16	28	300	4T16	14	250	4T16	8	250	4T16	4	31.37
40	9 × 5000	140	450	250	300	4T16	32	300	4T16	16	250	4T16	8	250	4T16	4	31.24
40	10 × 4500	120	450	250	250	4T16	36	250	4T16	8	250	4T16	18	250	4T16	4	29.49
40	11 × 4091	120	400	250	250	4T16	40	250	4T16	8	250	4T16	20	250	4T16	4	29.85
40	12 × 3750	100	400	250	250	4T16	44	250	4T16	8	250	4T16	22	250	4T16	4	28.15

f_{cu} (MPa)	$n_x \times L_x$ (mm)	Floors			Interior Columns			Edge Columns (*x*-direction)			Edge Columns (*y*-direction)			Corner Columns			Total Cost (USD/m²)
		t^{sl} (mm)	h^b (mm)	w^b (mm)	b^{cl} (mm)	Steel bars	No. of Columns	b^{cl} (mm)	Steel bars	No. of Columns	b^{cl} (mm)	Steel bars	No. of Columns	b^{cl} (mm)	Steel bars	No. of Columns	
45	7 × 6429	140	500	250	300	4T16	24	300	4T16	12	250	4T16	8	250	4T16	4	32.72
45	8 × 5625	140	450	250	250	4T18	28	250	4T16	14	250	4T16	8	250	4T16	4	32.17
45	9 × 5000	140	450	250	250	4T16	32	250	4T16	16	250	4T16	8	250	4T16	4	31.92
45	10 × 4500	120	400	250	250	4T16	36	250	4T16	8	250	4T16	18	250	4T16	4	30.22
45	11 × 4091	120	400	250	250	4T16	40	250	4T16	8	250	4T16	20	250	4T16	4	30.73
45	12 × 3750	100	400	250	250	4T16	44	250	4T16	8	250	4T16	22	250	4T16	4	28.98
50	7 × 6429	140	600	250	300	4T16	24	300	4T16	12	250	4T16	8	250	4T16	4	33.87
50	8 × 5625	140	450	250	250	4T16	28	250	4T16	14	250	4T16	8	250	4T16	4	32.89
50	9 × 5000	140	450	250	250	4T16	32	250	4T16	16	250	4T16	8	250	4T16	4	32.75
50	10 × 4500	120	400	250	250	4T16	36	250	4T16	8	250	4T16	18	250	4T16	4	31.01
50	11 × 4091	120	400	250	250	4T16	40	250	4T16	8	250	4T16	20	250	4T16	4	31.43
50	12 × 3750	100	400	250	250	4T16	44	250	4T16	8	250	4T16	22	250	4T16	4	29.68
55	7 × 6429	140	600	250	250	4T16	24	250	4T16	12	250	4T16	8	250	4T16	4	34.66
55	8 × 5625	140	450	250	250	4T16	28	250	4T16	14	250	4T16	8	250	4T16	4	33.83
55	9 × 5000	140	450	250	250	4T16	32	250	4T16	16	250	4T16	8	250	4T16	4	33.69
55	10 × 4500	120	400	250	250	4T16	36	250	4T16	8	250	4T16	18	250	4T16	4	31.93
55	11 × 4091	120	400	250	250	4T16	40	250	4T16	8	250	4T16	20	250	4T16	4	32.31
55	12 × 3750	100	400	250	250	4T16	44	250	4T16	8	250	4T16	22	250	4T16	4	30.48
60	7 × 6429	140	600	250	250	4T16	24	250	4T16	12	250	4T16	8	250	4T16	4	35.76
60	8 × 5625	140	450	250	250	4T16	28	250	4T16	14	250	4T16	8	250	4T16	4	34.73
60	9 × 5000	140	450	250	250	4T16	32	250	4T16	16	250	4T16	8	250	4T16	4	34.67
60	10 × 4500	120	450	250	250	4T16	36	250	4T16	8	250	4T16	18	250	4T16	4	32.95
60	11 × 4091	120	400	250	250	4T16	40	250	4T16	8	250	4T16	20	250	4T16	4	33.10
60	12 × 3750	100	400	250	250	4T16	44	250	4T16	8	250	4T16	22	250	4T16	4	31.27

Table S4. Summary of FS optimal results for different column spacing and concrete grade variants (case study 2).

f_{cu} (MPa)	$n \times L$ (mm)	Floors	Interior Columns			Edge Columns (x-direction)			Edge Columns (y-direction)			Corner Columns			Total Cost (USD/m²)
		t^{sl} (mm)	b^{cl} (mm)	Steel bars	No. of Columns	b^{cl} (mm)	Steel bars	No. of Columns	b^{cl} (mm)	Steel bars	No. of Columns	b^{cl} (mm)	Steel bars	No. of Columns	
25	4 × 7500	260	750	12T22	9	550	8T22	6	550	8T22	6	500	8T18	4	42.95
25	5 × 6000	200	600	12T18	16	450	8T18	8	450	8T18	8	400	8T18	4	35.48
25	6 × 5000	180	450	8T18	25	400	8T16	10	400	8T16	10	350	8T16	4	32.11
25	7 × 4286	160	400	8T16	36	350	8T16	12	350	8T16	12	300	4T16	4	30.31
25	8 × 3750	160	350	8T16	49	300	4T18	14	300	4T18	14	300	4T16	4	30.40
25	9 × 3333	160	350	8T16	64	300	4T16	16	300	4T16	16	300	4T16	4	31.55
35	4 × 7500	240	650	12T22	9	500	8T18	6	500	8T18	6	450	8T18	4	42.61
30	5 × 6000	200	550	8T22	16	450	8T18	8	400	8T18	8	400	8T16	4	35.76
30	6 × 5000	180	400	8T18	25	350	8T16	10	350	8T16	10	350	8T16	4	32.38
30	7 × 4286	160	400	8T16	36	350	8T16	12	350	8T16	12	300	4T16	4	30.88
30	8 × 3750	160	350	8T16	49	300	4T16	14	300	4T16	14	300	4T16	4	30.76
30	9 × 3333	160	300	4T22	64	300	4T16	16	300	4T16	16	300	4T16	4	31.58
35	4 × 7500	240	650	12T22	9	500	8T18	6	500	8T18	6	450	8T18	4	42.61
35	5 × 6000	200	500	8T18	16	400	8T16	8	400	8T16	8	350	8T16	4	35.23
35	6 × 5000	160	450	8T18	25	350	8T16	10	350	8T16	10	350	8T16	4	32.28
35	7 × 4286	160	350	8T16	36	350	8T16	12	350	8T16	12	300	4T16	4	30.95
35	8 × 3750	160	350	8T16	49	300	4T16	14	300	4T16	14	300	4T16	4	31.32
35	9 × 3333	160	300	4T18	64	300	4T16	16	300	4T16	16	300	4T16	4	30.95
40	4 × 7500	240	600	12T18	9	500	8T18	6	500	8T18	6	450	8T18	4	42.68
40	5 × 6000	200	450	8T18	16	400	8T16	8	400	8T16	8	350	8T16	4	35.62
40	6 × 5000	160	400	8T16	25	350	8T16	10	350	8T16	10	350	8T16	4	32.10
40	7 × 4286	160	350	8T16	36	300	4T16	12	300	4T16	12	300	4T16	4	30.64
40	8 × 3750	160	300	4T22	49	300	4T16	14	300	4T16	14	300	4T16	4	31.37
40	9 × 3333	160	300	4T16	64	300	4T16	16	300	4T16	16	300	4T16	4	31.04

f_{cu} (MPa)	$n \times L$ (mm)	Floors	Interior Columns			Edge Columns (x-direction)			Edge Columns (y-direction)			Corner Columns			Total Cost (USD/m²)
		t^{sl} (mm)	b^{cl} (mm)	Steel bars	No. of Columns	b^{cl} (mm)	Steel bars	No. of Columns	b^{cl} (mm)	Steel bars	No. of Columns	b^{cl} (mm)	Steel bars	No. of Columns	
45	4 × 7500	240	550	8T22	9	450	8T18	6	500	8T18	6	450	8T18	4	43.72
45	5 × 6000	200	450	8T18	16	400	8T16	8	400	8T16	8	350	8T16	4	36.70
45	6 × 5000	160	400	8T16	25	300	4T22	10	300	4T22	10	300	4T18	4	32.67
45	7 × 4286	160	350	8T16	36	300	4T16	12	300	4T16	12	300	4T16	4	31.53
45	8 × 3750	160	300	4T16	49	300	4T16	14	300	4T16	14	400	8T18	4	31.25
45	9 × 3333	160	300	4T16	64	300	4T16	16	300	4T16	16	300	4T16	4	31.97
50	4 × 7500	240	550	8T22	9	450	8T18	6	450	8T18	6	450	8T18	4	44.87
50	5 × 6000	200	450	8T18	16	400	8T16	8	400	8T16	8	350	8T16	4	37.60
50	6 × 5000	160	400	8T16	25	300	4T18	10	300	4T18	10	300	4T18	4	33.18
50	7 × 4286	160	350	8T16	36	300	4T16	12	300	4T16	12	300	4T16	4	32.43
50	8 × 3750	160	300	4T16	49	300	4T16	14	300	4T16	14	300	4T16	4	31.88
50	9 × 3333	160	300	4T16	64	300	4T16	16	300	4T16	16	300	4T16	4	32.90
55	4 × 7500	240	550	8T22	9	450	8T18	6	450	8T18	6	450	8T18	4	46.13
55	5 × 6000	200	450	8T18	16	400	8T16	8	350	8T16	8	400	8T16	4	38.61
55	6 × 5000	160	400	8T16	25	300	4T16	10	300	4T16	10	350	8T16	4	34.04
55	7 × 4286	160	300	4T22	36	300	4T16	12	300	4T16	12	300	4T16	4	32.97
55	8 × 3750	160	300	4T16	49	300	4T16	14	300	4T16	14	300	4T16	4	32.79
55	9 × 3333	160	300	4T16	64	300	4T16	16	300	4T16	16	300	4T16	4	33.83
60	4 × 7500	240	550	8T22	9	450	8T18	6	450	8T18	6	450	8T18	4	47.40
60	5 × 6000	200	450	8T18	16	400	8T16	8	350	8T16	8	400	8T16	4	39.68
60	6 × 5000	160	400	8T16	25	300	4T16	10	300	4T16	10	300	4T18	4	34.80
60	7 × 4286	160	300	4T16	36	300	4T16	12	300	4T16	12	300	4T16	4	32.90
60	8 × 3750	160	300	4T16	49	300	4T16	14	300	4T16	14	300	4T16	4	33.69
60	9 × 3333	160	300	4T16	64	300	4T16	16	300	4T16	16	300	4T16	4	34.76

Table S5. Summary of FSDP optimal results for different column spacing and concrete grade variants (case study 2).

f_{cu} (MPa)	$n \times L$ (mm)	Floors			Interior Columns			Edge Columns (*x*-direction)			Edge Columns (*y*-direction)			Corner Columns			Total Cost (USD/m²)
		t^{sl} (mm)	t^{drop} (mm)	S^{drop} (mm)	b^{cl} (mm)	Steel bars	No. of Columns	b^{cl} (mm)	Steel bars	No. of Columns	b^{cl} (mm)	Steel bars	No. of Columns	b^{cl} (mm)	Steel bars	No. of Columns	
25	4 × 7500	240	120	2550	600	12T22	9	500	8T22	6	500	8T22	6	450	8T16	4	43.06
25	5 × 6000	200	100	2200	500	8T18	16	450	8T18	8	450	8T22	8	400	8T16	4	36.95
25	6 × 5000	180	60	2400	450	8T18	25	400	8T16	10	400	8T22	10	350	8T16	4	34.07
25	7 × 4286	160	40	2100	400	8T16	36	350	8T16	12	350	8T22	12	300	4T16	4	32.16
25	8 × 3750	160	40	1750	350	8T16	49	350	8T16	14	300	4T22	14	300	4T16	4	31.86
25	9 × 3333	160	60	1150	350	8T16	64	300	4T16	16	300	4T22	16	300	4T16	4	32.81
30	4 × 7500	240	100	2750	600	12T18	9	500	8T18	6	500	8T22	6	450	8T16	4	42.78
30	5 × 6000	180	60	2000	500	8T18	16	400	8T18	8	400	8T22	8	350	8T16	4	36.49
30	6 × 5000	160	60	1800	400	8T18	25	350	8T16	10	350	8T22	10	300	4T16	4	33.64
30	7 × 4286	160	40	2100	400	8T16	36	350	8T16	12	350	8T22	12	300	4T16	4	32.76
30	8 × 3750	160	40	1750	350	8T16	49	300	4T16	14	300	4T22	14	300	4T16	4	31.97
30	9 × 3333	160	60	1150	300	4T22	64	300	4T16	16	300	4T22	16	300	4T16	4	32.78
35	4 × 7500	220	80	2600	550	8T22	9	450	8T18	6	450	8T22	6	450	8T16	4	41.99
35	5 × 6000	180	100	2200	450	8T18	16	400	8T16	8	400	8T22	8	350	8T16	4	36.53
35	6 × 5000	160	40	2450	400	8T16	25	350	8T16	10	350	8T22	10	350	8T16	4	33.50
35	7 × 4286	160	40	2100	350	8T16	36	300	4T18	12	300	4T22	12	300	4T16	4	31.78
35	8 × 3750	160	40	1750	350	8T16	49	300	4T16	14	300	4T22	14	300	4T16	4	32.56
35	9 × 3333	160	60	1150	300	4T16	64	300	4T16	16	300	4T22	16	300	4T16	4	31.69
40	4 × 7500	220	80	2600	500	8T18	9	500	8T18	6	450	8T22	6	500	8T16	4	42.40
40	5 × 6000	180	60	2250	450	8T18	16	400	8T16	8	400	8T22	8	350	8T16	4	37.06
40	6 × 5000	160	40	2450	400	8T16	25	350	8T16	10	350	8T22	10	350	8T16	4	34.08
40	7 × 4286	160	40	2100	350	8T16	36	300	4T16	12	300	4T22	12	300	4T16	4	32.26
40	8 × 3750	160	40	1550	300	4T22	49	300	4T16	14	300	4T22	14	300	4T16	4	32.66
40	9 × 3333	160	60	1150	300	4T16	64	300	4T16	16	300	4T22	16	300	4T16	4	32.28

f_{cu} (MPa)	$n \times L$ (mm)	Floors			Interior Columns			Edge Columns (x-direction)			Edge Columns (y-direction)			Corner Columns			Total Cost (USD/m²)
		t^{sl} (mm)	t^{drop} (mm)	S^{drop} (mm)	b^{cl} (mm)	Steel bars	No. of Columns	b^{cl} (mm)	Steel bars	No. of Columns	b^{cl} (mm)	Steel bars	No. of Columns	b^{cl} (mm)	Steel bars	No. of Columns	
45	4 × 7500	220	120	2600	500	8T18	9	450	8T18	6	450	8T22	6	450	8T16	4	43.58
45	5 × 6000	180	60	2600	450	8T18	16	400	8T16	8	350	8T22	8	350	8T16	4	37.77
45	6 × 5000	160	40	2500	350	8T16	25	350	8T16	10	350	8T22	10	350	8T16	4	34.73
45	7 × 4286	160	40	2100	350	8T16	36	300	4T16	12	300	4T22	12	300	4T16	4	33.20
45	8 × 3750	160	40	1550	300	4T16	49	300	4T16	14	300	4T22	14	300	4T16	4	32.31
45	9 × 3333	160	60	1150	300	4T16	64	300	4T16	16	300	4T22	16	300	4T16	4	33.24
50	4 × 7500	220	80	2600	450	8T18	9	450	8T18	6	450	8T22	6	450	8T16	4	44.58
50	5 × 6000	180	60	2000	400	8T16	16	400	8T16	8	400	8T22	8	400	8T16	4	38.73
50	6 × 5000	160	40	2450	400	8T16	25	300	4T18	10	300	4T22	10	300	4T16	4	34.88
50	7 × 4286	160	40	2100	350	8T16	36	300	4T16	12	300	4T22	12	300	4T16	4	34.15
50	8 × 3750	160	40	1550	300	4T16	49	300	4T16	14	300	4T22	14	300	4T16	4	33.24
50	9 × 3333	160	60	1150	300	4T16	64	300	4T16	16	300	4T22	16	300	4T16	4	34.21
55	4 × 7500	220	80	2600	450	8T18	9	450	8T18	6	450	8T22	6	450	8T16	4	45.78
55	5 × 6000	180	80	2000	400	8T16	16	350	8T16	8	350	8T22	8	350	8T16	4	39.33
55	6 × 5000	160	40	2500	350	8T16	25	300	4T18	10	300	4T22	10	300	4T16	4	35.49
55	7 × 4286	160	40	1700	300	4T18	36	300	4T16	12	350	8T22	12	350	8T16	4	35.36
55	8 × 3750	160	40	1550	300	4T16	49	300	4T16	14	300	4T22	14	300	4T16	4	34.18
55	9 × 3333	160	60	1150	300	4T16	64	300	4T16	16	300	4T22	16	300	4T16	4	35.17
60	4 × 7500	220	60	2600	450	8T18	9	400	8T18	6	450	8T22	6	450	8T16	4	46.82
60	5 × 6000	180	80	2000	400	8T16	16	350	8T16	8	400	8T22	8	400	8T16	4	40.54
60	6 × 5000	160	40	2500	350	8T16	25	300	4T16	10	300	4T22	10	400	8T16	4	36.55
60	7 × 4286	160	40	2150	300	4T16	36	300	4T16	12	300	4T22	12	300	4T16	4	34.80
60	8 × 3750	160	40	1550	300	4T16	49	300	4T16	14	300	4T22	14	300	4T16	4	35.11
60	9 × 3333	160	40	1150	300	4T16	64	300	4T16	16	300	4T22	16	300	4T16	4	36.14

Table S6. Summary of SS optimal results for different column spacing and concrete grade variants (case study 2).

f_{cu} (MPa)	$n \times L$ (mm)	Floors			Interior Columns			Edge Columns (*x*-direction)			Edge Columns (*y*-direction)			Corner Columns			Total Cost (USD/m²)
		t^{sl} (mm)	h^b (mm)	w^b (mm)	b^{cl} (mm)	Steel bars	No. of Columns	b^{cl} (mm)	Steel bars	No. of Columns	b^{cl} (mm)	Steel bars	No. of Columns	b^{cl} (mm)	Steel bars	No. of Columns	
25	4 × 7500	200	850	250	600	12T22	9	600	12T16	6	450	8T16	6	450	8T16	4	42.63
25	5 × 6000	160	600	250	500	8T18	16	500	8T16	8	350	8T16	8	350	8T16	4	37.46
25	6 × 5000	140	500	250	400	8T18	25	400	8T16	10	350	8T16	10	300	4T16	4	32.65
25	7 × 4286	120	400	250	350	8T16	36	350	8T16	12	300	4T16	12	300	4T16	4	31.00
25	8 × 3750	100	400	250	350	8T16	49	350	8T16	14	300	4T16	14	250	4T16	4	30.76
25	9 × 3333	100	400	250	300	4T22	64	300	4T16	16	250	4T16	16	250	4T16	4	31.81
30	4 × 7500	200	800	250	600	12T18	9	600	12T16	6	450	8T16	6	400	8T16	4	42.36
30	5 × 6000	160	600	250	500	8T18	16	500	8T16	8	350	8T16	8	300	4T16	4	37.77
30	6 × 5000	140	500	250	400	8T16	25	400	8T16	10	400	8T16	10	300	4T16	4	32.94
30	7 × 4286	120	400	250	350	8T16	36	350	8T16	12	300	4T16	12	250	4T16	4	31.40
30	8 × 3750	100	400	250	300	4T22	49	300	4T16	14	250	4T16	14	250	4T16	4	30.34
30	9 × 3333	100	400	250	300	4T16	64	300	4T16	16	250	4T16	16	250	4T16	4	30.91
35	4 × 7500	200	750	250	600	12T18	9	600	12T16	6	400	8T16	6	350	8T16	4	42.90
35	5 × 6000	160	650	250	450	8T18	16	450	8T16	8	350	8T16	8	300	4T16	4	38.11
35	6 × 5000	140	500	250	350	8T16	25	350	8T16	10	300	4T16	10	300	4T16	4	32.52
35	7 × 4286	120	400	250	350	8T16	36	350	8T16	12	300	4T16	12	250	4T16	4	31.96
35	8 × 3750	100	400	250	300	4T18	49	300	4T16	14	250	4T16	14	250	4T16	4	29.97
35	9 × 3333	100	400	250	300	4T16	64	300	4T16	16	250	4T16	16	250	4T16	4	31.49
40	4 × 7500	200	750	250	500	8T22	9	500	8T16	6	400	8T16	6	350	8T16	4	43.01
40	5 × 6000	160	650	250	400	8T18	16	400	8T16	8	350	8T16	8	300	4T16	4	38.38
40	6 × 5000	140	450	250	350	8T16	25	350	8T16	10	300	4T16	10	300	4T16	4	33.03
40	7 × 4286	120	400	250	350	8T16	36	350	8T16	12	250	4T16	12	250	4T16	4	32.42
40	8 × 3750	100	400	250	300	4T16	49	300	4T16	14	250	4T16	14	250	4T16	4	30.14
40	9 × 3333	100	400	250	300	4T16	64	300	4T16	16	250	4T16	16	250	4T16	4	32.06

f_{cu} (MPa)	$n \times L$ (mm)	Floors			Interior Columns			Edge Columns (*x*-direction)			Edge Columns (*y*-direction)			Corner Columns			Total Cost (USD/m²)
		t^{sl} (mm)	h^b (mm)	w^b (mm)	b^{cl} (mm)	Steel bars	No. of Columns	b^{cl} (mm)	Steel bars	No. of Columns	b^{cl} (mm)	Steel bars	No. of Columns	b^{cl} (mm)	Steel bars	No. of Columns	
45	4 × 7500	200	750	250	500	8T18	9	500	8T16	6	350	8T16	6	350	8T16	4	43.94
45	5 × 6000	160	550	250	400	8T16	16	400	8T16	8	300	4T16	8	350	8T16	4	39.05
45	6 × 5000	140	500	250	350	8T16	25	350	8T16	10	300	4T16	10	250	4T16	4	34.23
45	7 × 4286	120	400	250	300	4T18	36	400	8T16	12	300	4T16	12	350	8T16	4	32.72
45	8 × 3750	100	400	250	300	4T16	49	300	4T16	14	250	4T16	14	250	4T16	4	31.19
45	9 × 3333	100	400	250	300	4T16	64	300	4T16	16	250	4T16	16	250	4T16	4	33.11
50	4 × 7500	200	700	250	500	8T18	9	500	8T16	6	350	8T16	6	350	8T16	4	45.18
50	5 × 6000	160	550	250	400	8T16	16	400	8T16	8	300	4T16	8	300	4T16	4	39.92
50	6 × 5000	140	450	250	350	8T16	25	350	8T16	10	250	4T16	10	250	4T16	4	34.72
50	7 × 4286	120	400	250	300	4T16	36	300	4T16	12	250	4T16	12	250	4T16	4	32.48
50	8 × 3750	100	400	250	300	4T16	49	300	4T16	14	250	4T16	14	250	4T16	4	32.08
50	9 × 3333	100	400	250	250	4T16	64	300	4T16	16	250	4T16	16	250	4T16	4	33.42
55	4 × 7500	200	700	250	450	8T18	9	450	8T16	6	350	8T16	6	300	4T16	4	46.08
55	5 × 6000	160	550	250	350	8T16	16	350	8T16	8	300	4T16	8	300	4T16	4	40.68
55	6 × 5000	140	450	250	350	8T16	25	350	8T16	10	250	4T16	10	250	4T16	4	35.79
55	7 × 4286	120	400	250	300	4T16	36	300	4T16	12	250	4T16	12	250	4T16	4	33.50
55	8 × 3750	100	400	250	300	4T16	49	300	4T16	14	250	4T16	14	250	4T16	4	32.98
55	9 × 3333	100	400	250	250	4T16	64	250	4T16	16	250	4T16	16	250	4T16	4	34.23
60	4 × 7500	200	700	250	450	8T18	9	450	8T16	6	350	8T16	6	300	4T16	4	47.31
60	5 × 6000	160	550	250	350	8T16	16	350	8T16	8	300	4T16	8	250	4T16	4	41.62
60	6 × 5000	140	450	250	300	4T22	25	300	4T16	10	250	4T16	10	250	4T16	4	36.12
60	7 × 4286	120	400	250	300	4T16	36	300	4T16	12	250	4T16	12	250	4T16	4	34.42
60	8 × 3750	100	400	250	300	4T16	49	250	4T16	14	250	4T16	14	250	4T16	4	33.72
60	9 × 3333	100	400	250	250	4T16	64	250	4T16	16	250	4T16	16	250	4T16	4	35.14
